# Late‐in‐life treadmill training rejuvenates autophagy, protein aggregate clearance, and function in mouse hearts

**DOI:** 10.1111/acel.13467

**Published:** 2021-09-23

**Authors:** Jae Min Cho, Seul‐Ki Park, Rajeshwary Ghosh, Kellsey Ly, Caroline Ramous, Lauren Thompson, Michele Hansen, Maria Sara de Lima Coutinho Mattera, Karla Maria Pires, Maroua Ferhat, Sohom Mookherjee, Kevin J. Whitehead, Kandis Carter, Márcio Buffolo, Sihem Boudina, J. David Symons

**Affiliations:** ^1^ Nutrition and Integrative Physiology University of Utah Salt Lake City Utah USA; ^2^ School of Dentistry Sao Paulo State University Aracatuba Brazil; ^3^ Molecular Medicine Program University of Utah Salt Lake City Utah USA; ^4^ Division of Cardiovascular Medicine and Pediatric Cardiology University of Utah Salt Lake City Utah USA; ^5^ George E Wahlen VA Medical Center University of Utah Salt Lake City Utah USA

**Keywords:** aging, cardiac function, exercise, protein aggregates

## Abstract

Protein quality control mechanisms decline during the process of cardiac aging. This enables the accumulation of protein aggregates and damaged organelles that contribute to age‐associated cardiac dysfunction. Macroautophagy is the process by which post‐mitotic cells such as cardiomyocytes clear defective proteins and organelles. We hypothesized that late‐in‐life exercise training improves autophagy, protein aggregate clearance, and function that is otherwise dysregulated in hearts from old vs. adult mice. As expected, 24‐month‐old male C57BL/6J mice (old) exhibited repressed autophagosome formation and protein aggregate accumulation in the heart, systolic and diastolic dysfunction, and reduced exercise capacity vs. 8‐month‐old (adult) mice (all *p *< 0.05). To investigate the influence of late‐in‐life exercise training, additional cohorts of 21‐month‐old mice did (old‐ETR) or did not (old‐SED) complete a 3‐month progressive resistance treadmill running program. Body composition, exercise capacity, and soleus muscle citrate synthase activity improved in old‐ETR vs. old‐SED mice at 24 months (all *p *< 0.05). Importantly, protein expression of autophagy markers indicate trafficking of the autophagosome to the lysosome increased, protein aggregate clearance improved, and overall function was enhanced (all *p *< 0.05) in hearts from old‐ETR vs. old‐SED mice. These data provide the first evidence that a physiological intervention initiated late‐in‐life improves autophagic flux, protein aggregate clearance, and contractile performance in mouse hearts.

## Graphical abstract

Aged sedentary mice exhibit reduced cardiac: steady‐state autophagy; autophagic flux; protein clearance; redox balance; mitochondria quality; and function. If aged mice initiate exercise training at the proper intensity, frequency, and duration, each of these age‐associated cardiac disruptions is mitigated.

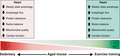

## 1 INTRODUCTION

The incidence of cardiovascular disease (CVD) is ~20%, ~50%, ~80%, and ~90% in individuals 18–44, 45–64, 65–79, and 80+ years of age, respectively (Benjamin et al., 2019). Treating cardiovascular complications associated with the aging demographic creates an enormous economic challenge to the healthcare system in particular, and society in general. New therapeutic targets need to be identified so that practical intervention strategies can be designed, optimized, and implemented. We sought to determine whether cardiac autophagy can be influenced positively by a well‐accepted lifestyle intervention strategy (i.e., regular physical activity) in a pre‐clinical model of primary aging.

Protein aggregates accumulate and organelles become damaged and/or dysfunctional during the process of aging. A progressive loss of protein quality control and autophagy contributes importantly in many organs to this age‐associated proteotoxicity and the subsequent decline in cellular function (Cuervo & Dice, 2000; Koga et al., 2011; Rubinsztein et al., 2011). Post‐mitotic cells with limited proliferative capacity such as cardiac myocytes are particularly reliant upon autophagy to maintain proteostasis and thereby preserve cardiac function during aging (Rubinsztein et al., 2011; Terman & Brunk, 2005). In support of this, age‐related cardiomyopathy is recapitulated in adult mice by cardiac‐specific Atg5 deletion (Taneike et al., 2010) and mTORC1 activation (Li et al., 2017; Taneike et al., 2016), whereas desmin‐related cardiomyopathy, characterized by the accumulation of cytotoxic misfolded proteins, is prevented by cardiac‐selective Atg7 overexpression (Bhuiyan et al., 2013).

Most literature indicates that primary aging precipitates myocardial dysfunction in C57BL/6J mice (Dai & Rabinovitch, 2009; Dai et al., 2009, 2012), but comparisons of cardiac autophagy between older and adult mice have not yielded consistent findings. Inconsistencies likely arise from conclusions being based solely upon steady‐state measures of autophagy including the quantification of MAP1LC3/LC3 (microtubule‐associated protein 1 light chain 3) and SQSTM1/p62 (Klionsky et al., 2021). However, because autophagy is a highly dynamic process, it is best practice to pharmacologically block the turnover of these proteins to most accurately quantify the scale of autophagosome formation, that is, autophagic flux. This methodological approach has been implemented once in aged mice (Wu et al., 2016) and once in cardiomyocytes isolated from older rats (Ma et al., 2017). Both studies provided support for an age‐associated repression of cardiac autophagic flux. Here, we substantiated these observations using chloroquine, and additionally showed accrual of ubiquitinated proteins and protein aggregates in the myocardium, oxidative stress, impaired mitochondrial quality, cardiac dysfunction, and reduced exercise capacity, in 24‐month‐ vs. 8‐month‐old animals. These findings allowed us to test our primary hypothesis that late‐in‐life exercise training re‐establishes autophagic flux to an extent that improves protein clearance, redox status, and cardiac function.

A growing area of research inquiry is whether upregulating the process of autophagy has therapeutic benefit. For example, late‐in‐life interventions that increase autophagy such as supplementation with the natural polyamine spermidine (Eisenberg et al., 2016), caloric restriction (Sheng et al., 2017), and mTORC1 inhibition using rapamycin (Flynn et al., 2013) lessen age‐associated cardiac dysfunction in C57BL/6J mice. An alternative or complementary approach with potential to improve cardiac autophagy and attenuate the aging‐associated decline in cardiac function is dynamic exercise. In this regard, He et al. observed that an acute bout of treadmill running elevates protein indexes of autophagy in murine hearts (He et al., 2012), and Bhuiyan et al. reported that autophagy, protein clearance, and function improved in hearts from mice with desmin‐related cardiomyopathy that did vs. did not have long‐term access (i.e., 6‐month) to wheel running (Bhuiyan et al., 2013). The potential for a physiological maneuver, that is, late‐in‐life exercise training, to rejuvenate cardiac autophagy has not been investigated. Here, we present what we believe to be the first report that exercise capacity, autophagic flux, protein aggregate clearance, redox balance, mitochondrial quality, and cardiac function improve in hearts from older mice that complete a progressive, resistance treadmill running program vs. animals that do not train.

## 2 RESULTS

### 2.1 Hearts from older mice display repressed autophagic flux, accumulation of ubiquitinated proteins, and oxidative stress

**FIGURE 1:**
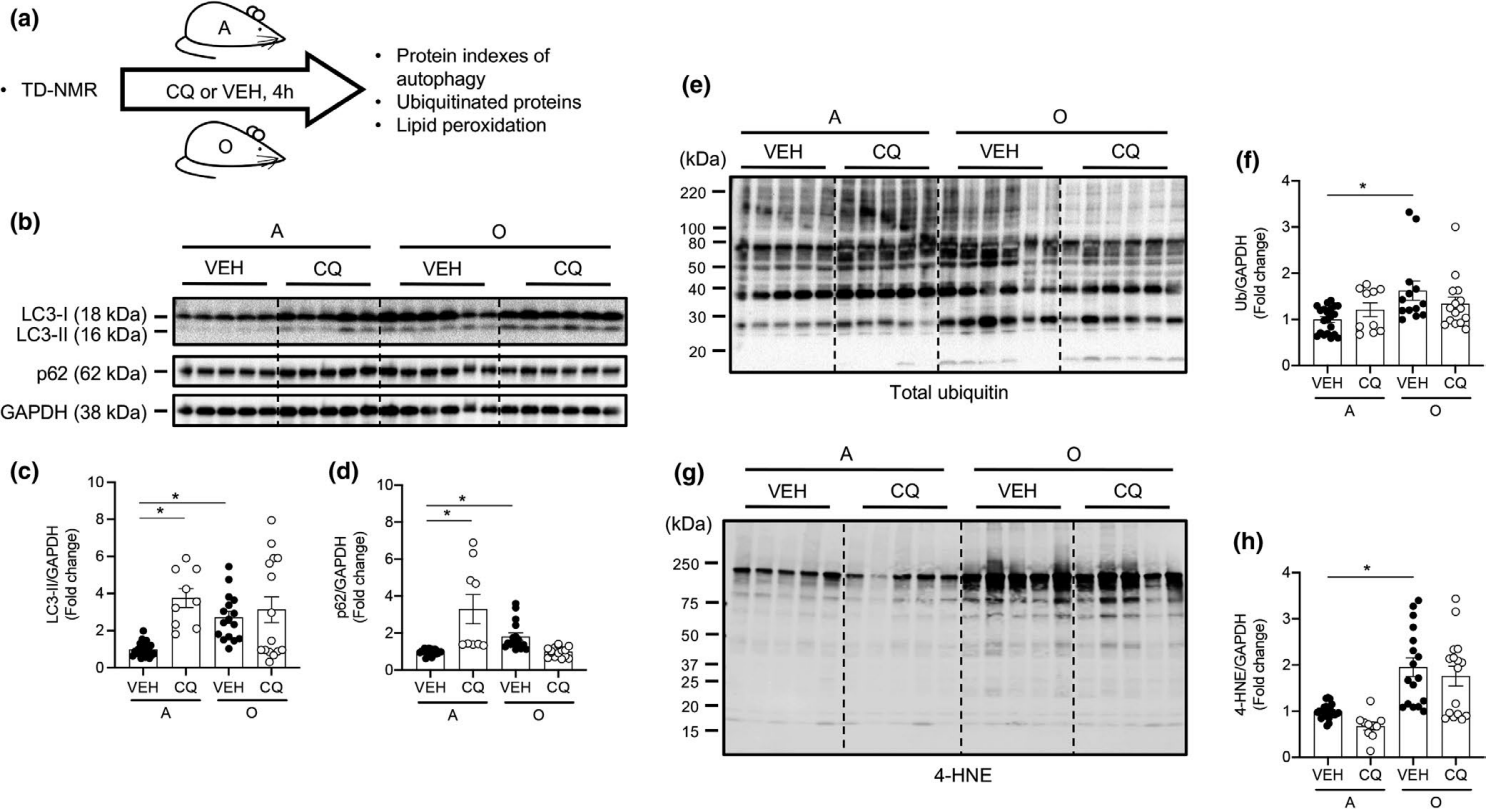
Hearts from old mice display impaired autophagic flux, accumulation of ubiquitinated proteins, and oxidative stress. (a) Body composition was assessed using TD‐NMR in adult (A; 8‐mo) and older (O; 24‐mo) mice. Vehicle (VEH; phosphate‐buffered saline, PBS) or chloroquine (CQ; 75 mg/kg lean muscle mass) was administered to A and O mice and tissues were obtained 4 h later. Representative images (b, e, g) and mean data ± standard error are shown for LC3‐II (c), p62 (d), poly‐ubiquitin (total ubiquitin; f), and 4‐hydroxy‐2‐nonenal (4‐HNE; h). LC3‐II and p62 were greater in hearts from O vs. A mice treated with VEH. In A mice, LC3‐II and p62 increased further in CQ vs. VEH‐treated cohorts, whereas these endpoints were similar in O mice treated with CQ. These findings demonstrate that autophagic flux is robust in A but not O mice. Total ubiquitin and 4‐HNE were elevated in hearts from O vs. A mice, but CQ did not alter responses in either group. For panels (c, d), *n* = 9–24, *p < 0.05 vs. A‐VEH mice. For (f) and (h), *n* = 10–21, *p < 0.05 vs. A‐VEH. For panels (c, d, f, h), data are expressed as fold change relative to values obtained from A‐VEH mice

The impact of aging on cardiac autophagy in pre‐clinical murine models is not uniform. Further, few studies have assessed the influence of aging on different steps involved in the process of myocardial autophagy (Ma et al., 2017; Wu et al., 2016). We sought clarity concerning the influence of aging on steady‐state autophagy and autophagic flux in the murine heart. Because lean mass is likely to change in an age‐associated and/or exercise‐training‐related manner, adult and older male C57BL/6J mice completed TD‐NMR analyses to determine body composition. This allowed chloroquine (CQ) to be administered at a dose based on lean body mass (Figure 1a) (Pires et al., 2017). Twenty‐four h after TD‐NMR, CQ (75 mg IP/g lean body mass) or vehicle‐control (phosphate‐buffered saline; VEH) was administered to both groups. After 4 h, hearts were collected from isoflurane‐anesthetized mice (Gottlieb et al., 2015; Klionsky et al., 2021; Pires et al., 2017). This CQ regimen is referred to in Figure 1a as “4 h.” We hypothesized that autophagic flux would be impaired in hearts from older vs. adult mice, and that this would associate with accrual of ubiquitinated proteins and heightened oxidative stress. Regarding steady‐state autophagy, representative images (Figure 1b) and mean data indicate protein expression of LC3‐I:GAPDH (Figure S1a), LC3‐II:GAPDH (Figure 1b, c), and p62:GAPDH (Figure 1b, d) was higher (*p* < 0.05), and Atg3:GAPDH was lower (Figure S1c, d; *p* < 0.05), in hearts from old‐VEH vs. adult‐VEH mice (histogram 3 vs. 1), whereas LC3‐II:LC3‐I, Atg5:GAPDH, and Atg7:GAPDH (Figure S1b, e, f) were similar between groups. Regarding mRNA expression, Atg3 was lower and p62 was higher (Figure S2, both *p* < 0.05) in hearts from old vs. adult mice, whereas LC3B mRNA expression was similar between groups (Figure S2). These findings were concurrent with accrual of ubiquitinated proteins (Figure 1e, f) and elevated 4‐hydroxy‐2‐nonenal (4‐HNE; Figure 1g, h; both *p* < 0.05). mRNA expression of superoxide dismutase (SOD) 2 trended higher (*p* = 0.06) and catalase was lower (*p *< 0.05) in hearts from old vs. adult mice, whereas SOD1 was similar between groups (Figure S2). Collectively, these findings strongly suggest that steady‐state autophagy is attenuated in hearts from old vs. adult mice.

It is not conclusive whether aging impairs autophagosome formation, trafficking of the autophagosome to the lysosome, and/or lysosomal degradation of the autophagosome in the heart. We addressed this by examining expression of autophagy proteins in the presence of chloroquine, which inhibits autophagosome‐lysosome fusion. Representative images (Figure 1b) and mean data indicate protein expression of LC3‐I:GAPDH (Figure S1a), LC3‐II:GAPDH and p62:GAPDH (Figure 1b–d) was higher (*p* < 0.05) in hearts from adult‐CQ vs. adult‐VEH mice (histogram 2 vs. 1), whereas LC3‐II: LC3‐I was not affected (Figures 1b, S1b). Elevated (*p* < 0.05) LC3‐II:GAPDH and p62:GAPDH in old‐VEH vs. adult‐VEH mice (histogram 3 vs. 1) did not increase further in older mice treated with CQ (histogram 3 vs. 4; Figure 1b–d), supporting the hypothesis that cardiac autophagic flux is impaired by aging. Neither protein expression of Atg3, Atg5, and Atg7 (Figure S1d‐f), nor mRNA expression of Atg3, p62, LC3B, SOD1, SOD2, and catalase (Figure S2) displayed by VEH‐treated mice were impacted by CQ in either group (data not shown).

### 2.2 Hearts from older mice display impaired systolic and diastolic function

**FIGURE 2:**
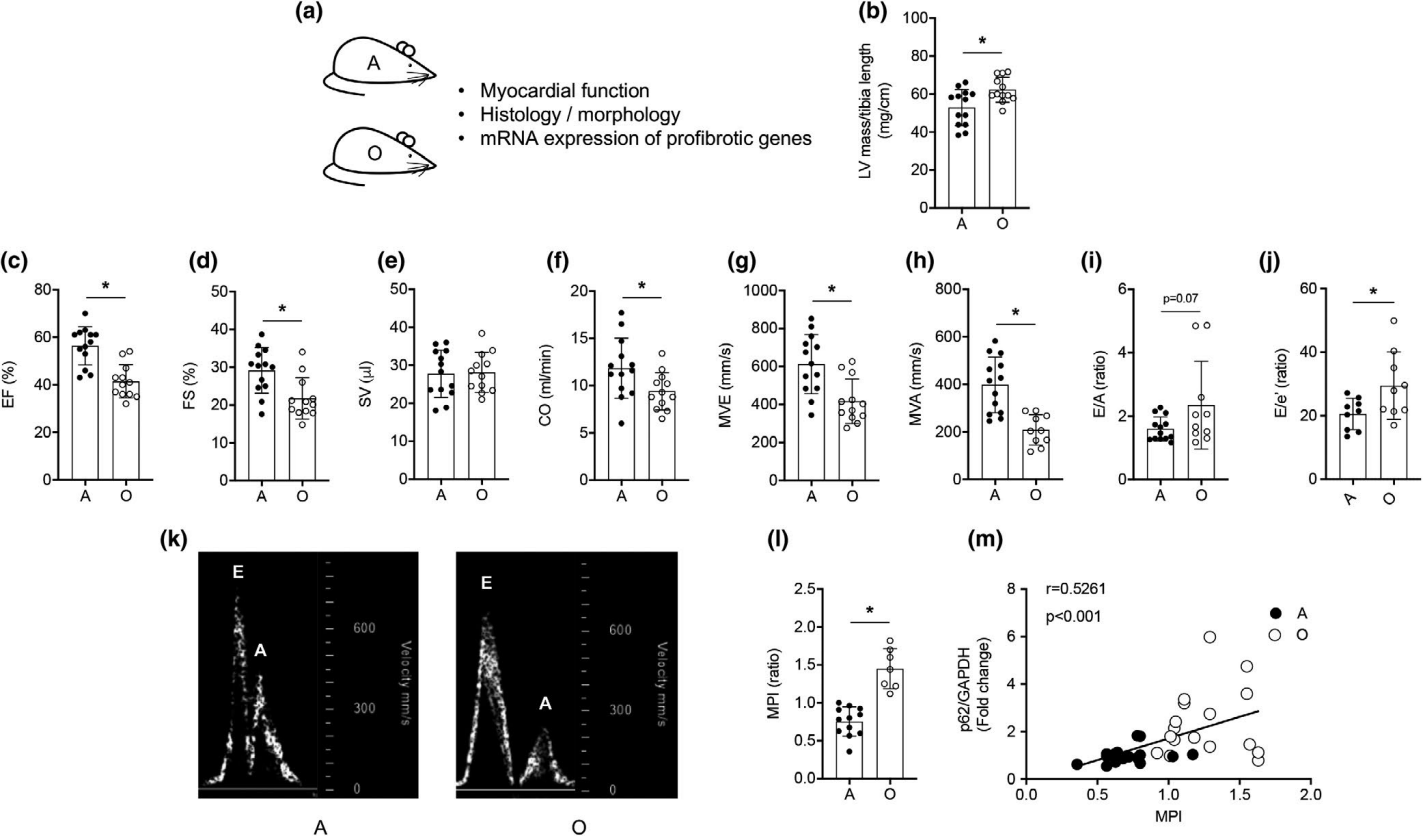
Cardiac function is impaired in older vs. adult mice. (a) Transthoracic echocardiography was performed on adult (A; 8‐mo) and older (O; 24‐mo) mice. Mean data ± standard error are shown (b‐j, l). Left ventricular (LV) mass normalized to tibial length was greater in O vs. A animals (b). Ejection fraction (EF, %; c), fractional shortening (FS, %; d), cardiac output (CO, ml/min; f), passive diastolic filling (MVE, mm/s; g), active diastolic filling (MVA, mm/s; h), and an estimate of end‐diastolic filling pressure (E/e’, ratio; j) were impaired in O vs. A mice, whereas stroke volume (SV, μl; e) and the E/A ratio (i) were not different between groups. These data, together with our observation that the myocardial performance index (MPI, l) is greater in hearts from O vs. A mice, indicate that systolic and diastolic dysfunction exists in O vs. A mice. (k) Representative images of blood flow velocity obtained during the assessment of MVE and MVA from both groups. (m) The correlation between protein expression of p62/GAPDH and MPI was strong in hearts from A and O mice. For (b–j, l), *n* = 9–13; for (m), *n* = 16–17. For (b–j, l), *p < 0.05 vs. A

While some discrepancies exist, the balance of available literature indicates that primary aging precipitates cardiac dysfunction in C57BL/6J mice (Dai & Rabinovitch, 2009; Dai et al., 2009, 2012). We hypothesized that aging‐associated cardiac dysfunction exists (Figure 2a). Compared to adult mice, older animals displayed greater left ventricular (LV) mass/tibia length (Figure 2b), lower ejection fraction (EF), fractional shortening (FS), cardiac output (CO; Figure 2c, d, f), and greater left ventricular internal dimension in systole (LVIDs), end‐systolic volume (ESV), and end‐diastolic volume (EDV; Figure S3a, c, d; all *p *< 0.05) indicating LV hypertrophy and systolic dysfunction exist in hearts from aged animals. Further, older mice exhibited diastolic dysfunction, for example, lower mitral valve early (MVE) and late (MVA) wave velocity (Figure 2g, h, k; *p* < 0.05), a trend toward an elevated E/A ratio (Figure 2i, k; *p *= 0.07), and a higher E/e’ ratio (an estimate of end‐diastolic pressure; Figure 2j, k; *p* < 0.05). The myocardial performance index (MPI) indicates overall LV function. MPI was elevated (i.e., function became worse) in hearts from older vs. adult mice (Figure 2l; *p* < 0.05). Of note, the accrual of p62:GAPDH (indicating repressed autophagy) associated (*p* < 0.001) with increased MPI (worsening of cardiac function; Figure 2m). Heart rate (beats/min) was not different between adult (406 ± 19) and older (378 ± 14) mice during the assessment of cardiac function.

Although type 1 collagen staining indicated increased fibrosis in hearts from older vs. adult mice, cardiomyocyte area was not affected (Figure S4a–c). Of the profibrotic genes that were assessed, mRNA expression of fibrillin (Fbn) 1 and transforming growth factor‐β (Tgfb) 2 were elevated (*p* < 0.05), and a trend existed for increased connective tissue growth factor (Ctgf; *p *= 0.07) in hearts from older vs. adult mice, whereas Fbn2 and Tgfb1 were not different between groups (Figure S4d).

### 2.3 Exercise training is efficacious in adult and older mice

**FIGURE 3:**
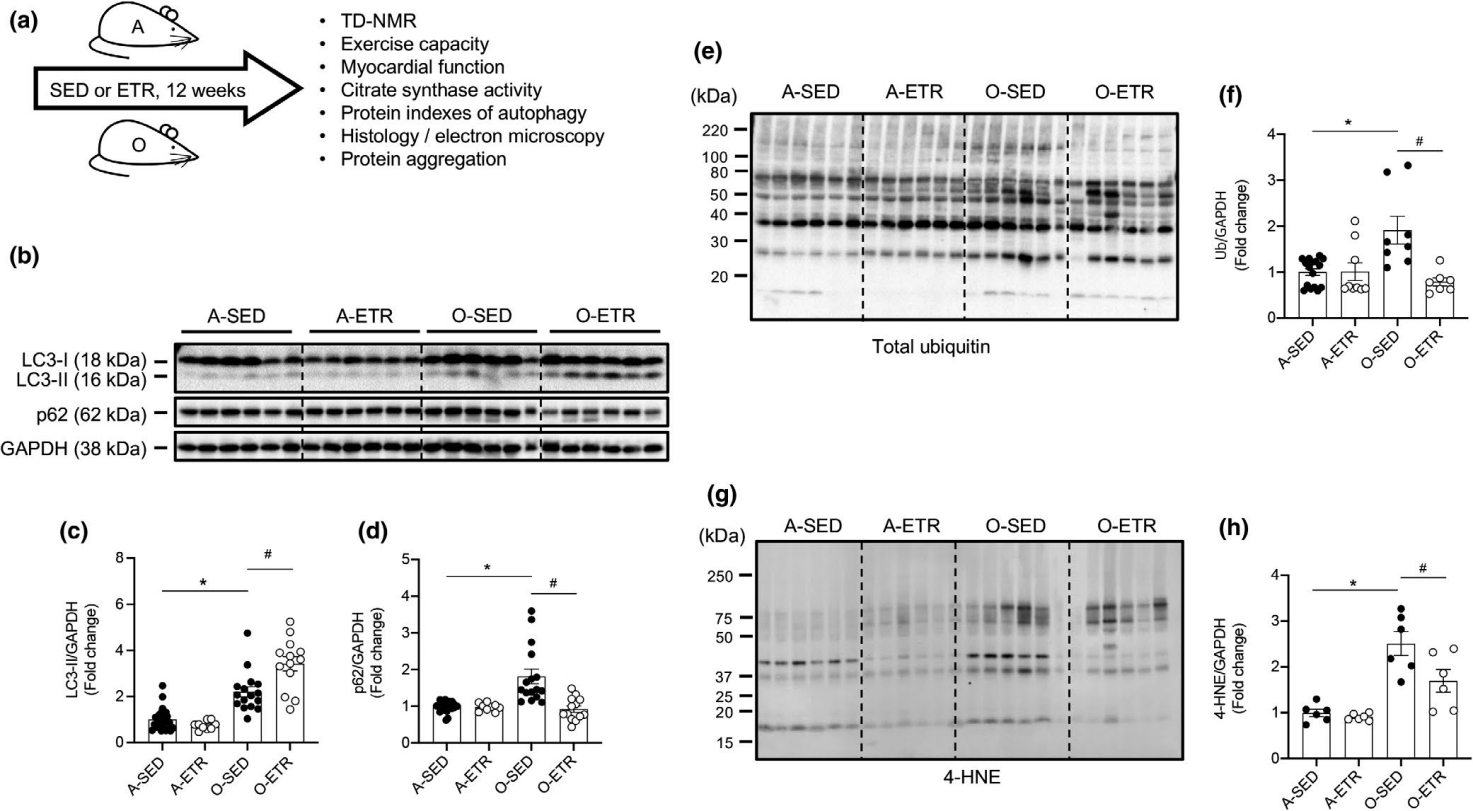
Late‐in‐life exercise training improves steady‐state autophagy, clearance of ubiquitinated proteins, and oxidative stress in mouse hearts. (a) Adult (A, 5 mo) and older (O, 21 mo) mice did (ETR) or did not (SED) complete 12 weeks of treadmill running. At least 24 h following the last exercise bout, A (8 mo) and O (24 mo) hearts were excised and prepared for immunoblotting. Representative images (b, e, g) and mean data ± standard error are shown for LC3‐II (c), p62 (d), total ubiquitin (f), and 4‐HNE (h). LC3‐II (c) and p62 (d) were greater in hearts from O‐SED vs. A‐SED mice. While no differences existed between A‐SED and A‐ETR mice, LC3‐II increased (c) and p62 decreased (d) in hearts from O‐ETR vs. O‐SED mice. Total ubiquitin (f) and 4‐HNE (h) were elevated in hearts from O‐SED vs. A‐SED mice. Although no differences existed between A‐SED and A‐ETR mice, total ubiquitin (f) and 4‐HNE (h) decreased in hearts from O‐ETR vs. O‐SED mice. For panels (c, d), *n* = 9–24, *p < 0.05 vs. A‐SED mice. For (f) and (h), *n* = 6–15, *p < 0.05 vs. A‐SED. For panels (c, d, f, h), data are expressed as fold change relative to values obtained from A‐SED mice

Our findings of repressed autophagic flux (Figure 1, Figure S1), ubiquitinated protein accrual and heightened oxidative stress (Figure 1), and impaired function (Figure 2) in hearts from older vs. adult mice were anticipated. Substantiating these observations was necessary to test the hypothesis that exercise training lessens the age‐associated disruptions. Adult mice did (adult‐ETR) or did not (adult‐SED) complete progressive resistance treadmill training from 5 to 8 months of age. Likewise, older mice did (old‐ETR) or did not (old‐SED) train from 21 to 24 months of age (Figure 3a). As expected, fat mass and body mass were greater (Figure S5a, b; *p* < 0.05), whereas total workload capacity and soleus muscle citrate synthase (CS) enzyme activity were less (Figure S5e, f; *p* < 0.05), in old‐SED vs. adult‐SED mice (histogram 3 vs. 1; (Table S1). Evidence for an exercise‐training effect included: (i) reduced fat mass (Figure S5b), and increased total workload capacity (Figure S5e) and CS activity (Figure S5f), in adult (histogram 2 vs. 1) and older (histogram 4 vs. 3) mice (*p* < 0.05 for all).

### 2.4 Exercise training improves steady‐state autophagy in hearts from older mice

Substantiating findings in Figure 1 that steady‐state autophagy is impaired in hearts from older vs. adult mice, representative images (Figure 3b) and mean data indicate protein expression of LC3I:GAPDH (Figures 3b, S6a), LC3II:GAPDH (Figure 3b, c), and p62:GAPDH (Figure 3b, d) was higher (*p* < 0.05), and Atg3:GAPDH (Figure S6c, d) was lower (*p* < 0.05), in hearts from older‐SED vs. adult‐SED mice (histogram 3 vs. 1). Regarding exercise‐training, indexes of cardiac autophagy were similar between adult‐ETR and adult‐SED mice (histogram 2 vs. 1; Figures 3 and S6). In contrast, LC3‐I:GAPDH (Figures 3b, S6a) and p62:GAPDH (Figure 3b, d) were less (*p* < 0.05), and LC3‐II:GAPDH (Figure 3b, c) and Atg3:GAPDH protein expression (Figure S6c, d) were greater (*p* < 0.05), in hearts from old‐ETR vs. old‐SED mice (histogram 4 vs. 3). mRNA expression (Figure S7) of Atg3 was higher and p62 was lower (both *p* < 0.05) in hearts from old‐ETR vs. old‐SED mice, whereas LC3B was similar between groups. Collectively, these findings indicate that late‐in‐life exercise training improves steady‐state autophagy in mouse hearts.

### 2.5 Exercise training improves clearance of ubiquitinated proteins and oxidative stress in hearts from older mice

Bolstering our findings shown in Figure 1e‐h, ubiquitinated proteins (Figure 3e, f) and indexes of lipid peroxidation (Figure 3g, h), were elevated (*p* < 0.05) in hearts from older‐VEH vs. adult‐VEH mice (histogram 3 vs. 1). Importantly, each age‐associated disruption was lessened (*p* < 0.05) in older‐ETR vs. older‐SED mice (histogram 4 vs. 3; Figure 3e–h). Adult mice were refractory to the effects of exercise training concerning these endpoints (Figure 3e–h; histogram 1 vs. 2). mRNA expression of SOD1, SOD2, and catalase were elevated by late‐in‐life exercise training (Figure S7).

### 2.6 Exercise training improves autophagic flux in hearts from older mice

**FIGURE 4:**
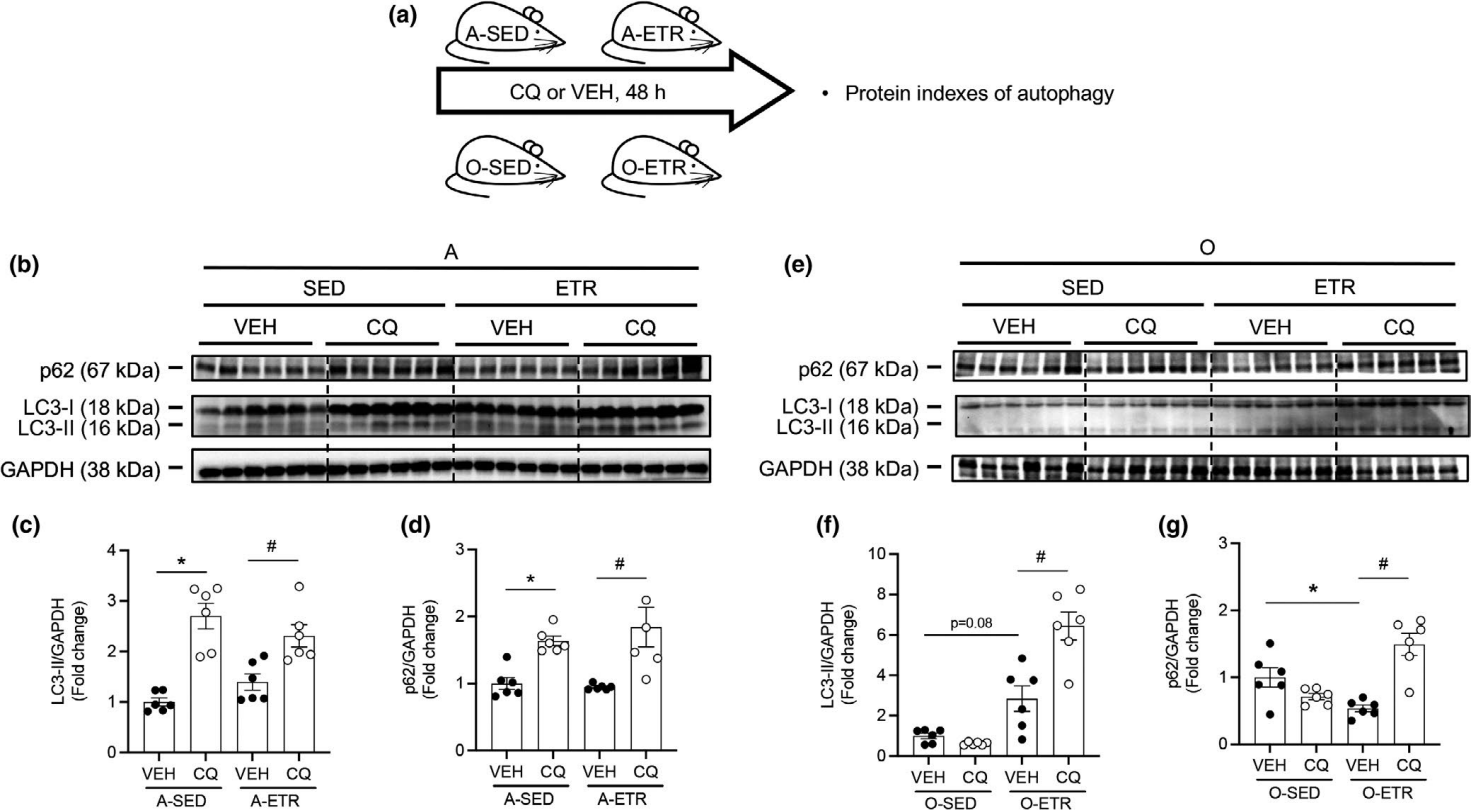
Late‐in‐life exercise training improves steady‐state autophagy and autophagic flux in mouse hearts. (a) Adult (A, 5 mo) and older (O, 21 mo) mice did (ETR) or did not (SED) complete 12 wk of treadmill running. Mice were treated with VEH or CQ 48, 24, and 4 h prior to tissue collection. Representative images (b, e) and mean data ± standard error are shown for LC3‐II (c, f) and p62 (d, g). In A hearts treated with VEH (b–d), ETR did not influence steady‐state LC3‐II or p62. Robust CQ‐evoked increases in LC3‐II and p62 observed in A hearts were similar regardless of exercise training (b–d). In O hearts treated with VEH, (e–g), LC3‐II trended upwards (*p *= 0.08) and p62 decreased in response to ETR. These data indicate that late‐in‐life exercise training improves steady‐state autophagy. While CQ did not influence LC3‐II or p62 in hearts from O‐SED mice (e–g), significant accumulation of LC3‐II (f) and p62 (g) was observed in hearts from O‐ETR mice. These findings indicate late‐in‐life exercise training improves autophagic flux in mouse hearts. For (b–g), *n* = 6. For (c, d), *p < 0.05 vs. A‐SED‐VEH; #*p *< 0.05 vs. A‐ETR‐VEH. For (f, g), *p < 0.05 vs. O‐SED‐VEH; #*p *< 0.05 vs. O‐ETR‐VEH. For (c, d, f, g), data are expressed as fold change relative to values obtained from A‐SED‐VEH mice or O‐SED‐VEH mice

Here, we tested whether exercise training improves the age‐associated repression of autophagic flux observed in Figure 1. Because exercise training did not influence steady‐state autophagy in adult mice (Figure 3), procedures displayed in Figure S8a were completed using old mice. Recapitulating results shown in Figure 3, LC3‐II:GAPDH accumulation (Figure S8b, c) and p62:GAPDH degradation (Figure S8b, d) was greater in (*p* < 0.05) in old‐ETR‐VEH vs. old‐SED‐VEH mice; histogram 3 vs. 1). As anticipated, and supporting results shown in Figure 1 for older mice, neither LC3‐II:GAPDH (Figure S8b, c) nor p62:GAPDH (Figure S8b, d) responded to CQ treatment in old‐SED animals (Figure S8b‐d; histogram 2 vs. 1). Notably, accrual of p62:GAPDH (Figure S8b, d) and Atg3: GAPDH (Figure S9b, c; both *p* < 0.05) occurred in the presence vs. the absence of 4 h CQ in old‐ETR animals (histogram 4 vs. 3). LC3‐I:GAPDH (Figure S8b, e), LC3‐II:LC3‐I (Figure S8b, f), Atg5:GAPDH (Figure S9b, d), and Atg7:GAPDH (Figure S9b, e) were not influenced by 4 h CQ treatment. Although p62:GAPDH responses to CQ suggest that autophagic flux was improved by late‐in‐life exercise training, LC3‐II:GAPDH (Figure S8b, c) results were not congruent with this interpretation. These findings, together with earlier results that 4 h CQ did not increase the LC3‐II:LC3‐I ratio in hearts from A mice (Figure S1b; histogram 2 vs. 1), prompted us to repeat these experiments using a longer duration of CQ treatment, that is, 48 h, achieved by CQ administration to the same mouse 48 h (30 mg/kg), 24 h (30 mg/kg), and 4 h (50 mg/kg) prior to tissue collection for immunoblotting. This CQ regimen is referred to in Figures 4a and S10a as “48h.” In addition, we included adult mice because even though exercise training does not influence steady‐state autophagy in this group (Figure 3), autophagic flux could be impacted.

Results that hearts from adult mice are refractory to exercise training concerning autophagy indexes shown earlier in Figure 3 were recapitulated here (Figure 4b–d; histogram 1 vs. 3). In contrast, substantiating findings shown in Figure 3 for older mice, LC3‐II:GAPDH trended higher (Figure 4e, f; *p *= 0.08) and p62:GAPDH decreased (Figure 4e–g; *p* < 0.05), in hearts from old‐ETR vs. old‐SED mice (histogram 1 vs. 3). Regarding autophagic flux in adult hearts, 48 h CQ evoked robust elevations in LC3‐II:GAPDH, p62:GAPDH (Figure 4b–d), and LC3‐II: LC3‐I (Figures 4b, S10c), in SED (histogram 1 vs. 2) and ETR (histogram 3 vs. 4) mice. Of most importance to our study, 48 h CQ increased LC3‐II:GAPDH and p62:GAPDH (Figure 4e–f) in hearts from old‐ETR (histogram 3 vs. 4) but not old‐SED mice (histogram 1 vs. 2). These findings, taken together with those from Figures 1, 3, and S8, S9, and S10, strongly support that autophagic flux that is otherwise repressed by aging can be rejuvenated by late‐in‐life exercise training.

### 2.7 Exercise training improves cardiac function in older mice

**FIGURE 5:**
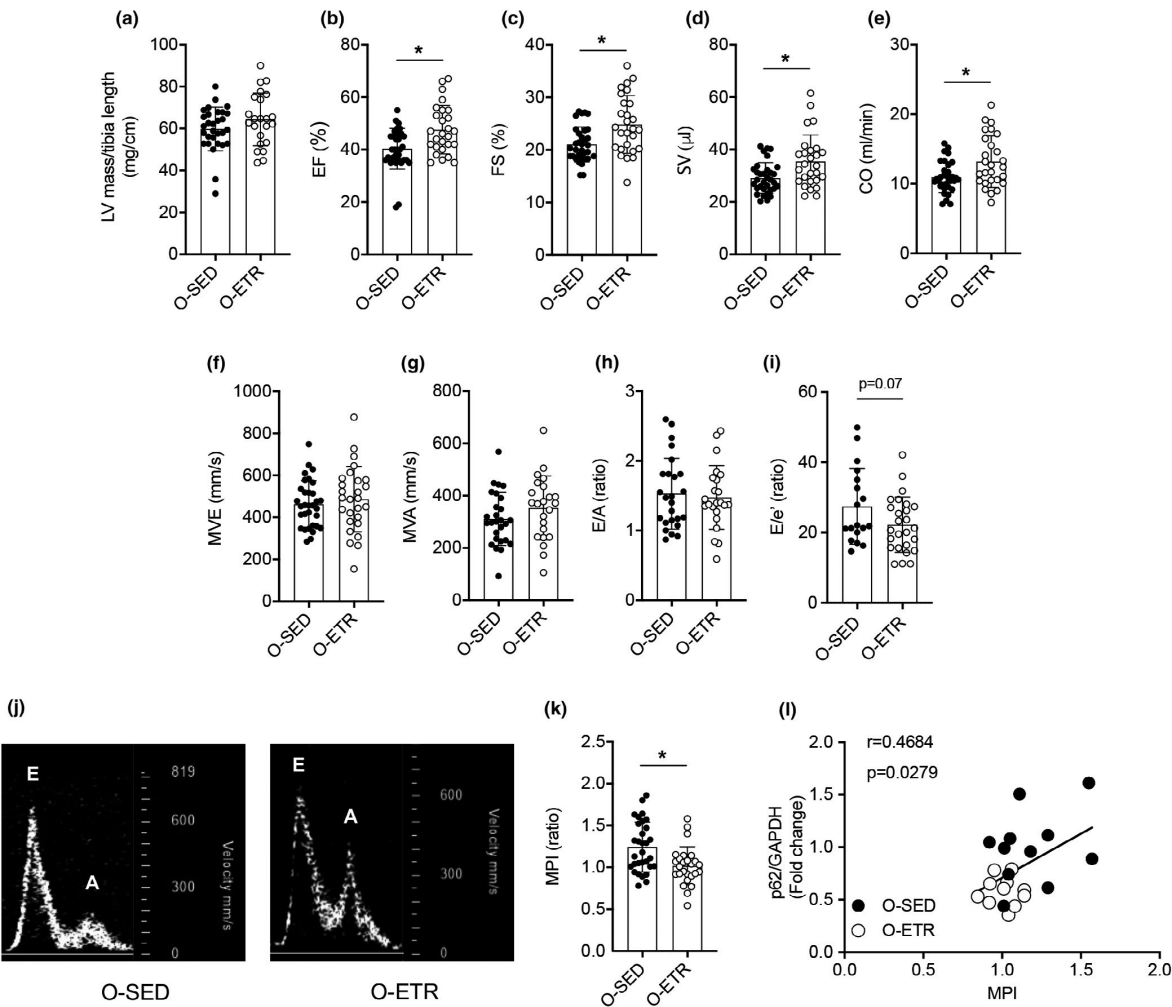
Late‐in‐life exercise training improves cardiac function in mice. Older (O, 21 mo) mice did (ETR) or did not (SED) complete 12 wk of treadmill running. At least 24 h following the last exercise bout, transthoracic echocardiography was completed on O‐SED and O‐ETR mice (24 mo). Mean data ± standard error are shown (a–i, k). Left‐ventricular mass normalized to tibia length was not different between groups (a). Ejection fraction (EF,%; b), fractional shortening (FS, %; c), stroke volume (SV, μl; d), and cardiac output (μl/min, e) were greater in O‐ETR vs. O‐SED mice, whereas passive diastolic filling (MVE, mm/s, f), active diastolic filling (MVA, mm/s, g), the MVA/MVE ratio (E/A, h), and an estimate of diastolic filling pressure (E/e’, ratio; i) were not different between groups. (j) Representative images of blood flow velocity obtained during the assessment of MVE and MVA from both groups. The myocardial performance index (MPI, k) was less in O‐ETR vs. O‐SED mice, indicating that function improved in trained mice. (l) The correlation between protein expression of p62/GAPDH and MPI was strong in hearts from O‐SED and O‐ETR mice. For panels (a–i) and (k), *n* = 18–33, *p < 0.05 vs. O‐SED. For (l), *n* = 11. Data are expressed as mean ± SEM

Next, we determined whether late‐in‐life, training‐induced improvements in steady‐state autophagy and autophagic flux associate positively with cardiac function. While LV mass/tibia length did not change (Figure 5a), indexes of systolic (Figure 5b–e) and overall LV function (i.e., MPI; Figure 5k) improved (*p* < 0.05), whereas estimates of diastolic function were unchanged (Figures 5f–j and S11), in old‐ETR vs. old‐SED mice. Importantly, in older mice, the training‐evoked increase in p62 protein degradation (indicating greater autophagy; Figures 3b, d; 4e, g) associated positively (*p* < 0.028) with the training‐induced lowering of MPI (indicating greater LV function; Figure 5l). A similar pattern of results was observed in adult‐ETR vs. adult‐SED animals (Figure S12, S13). Collagen type 1 area (Figure S14a, b), cardiomyocyte area (Figure S14a, c), and mRNA expression of profibrotic genes (Figure S14d) were similar between old‐SED and old‐ETR mice.

### 2.8 Exercise training improves protein aggregate removal and mitochondrial quality in hearts from older mice

**FIGURE 6:**
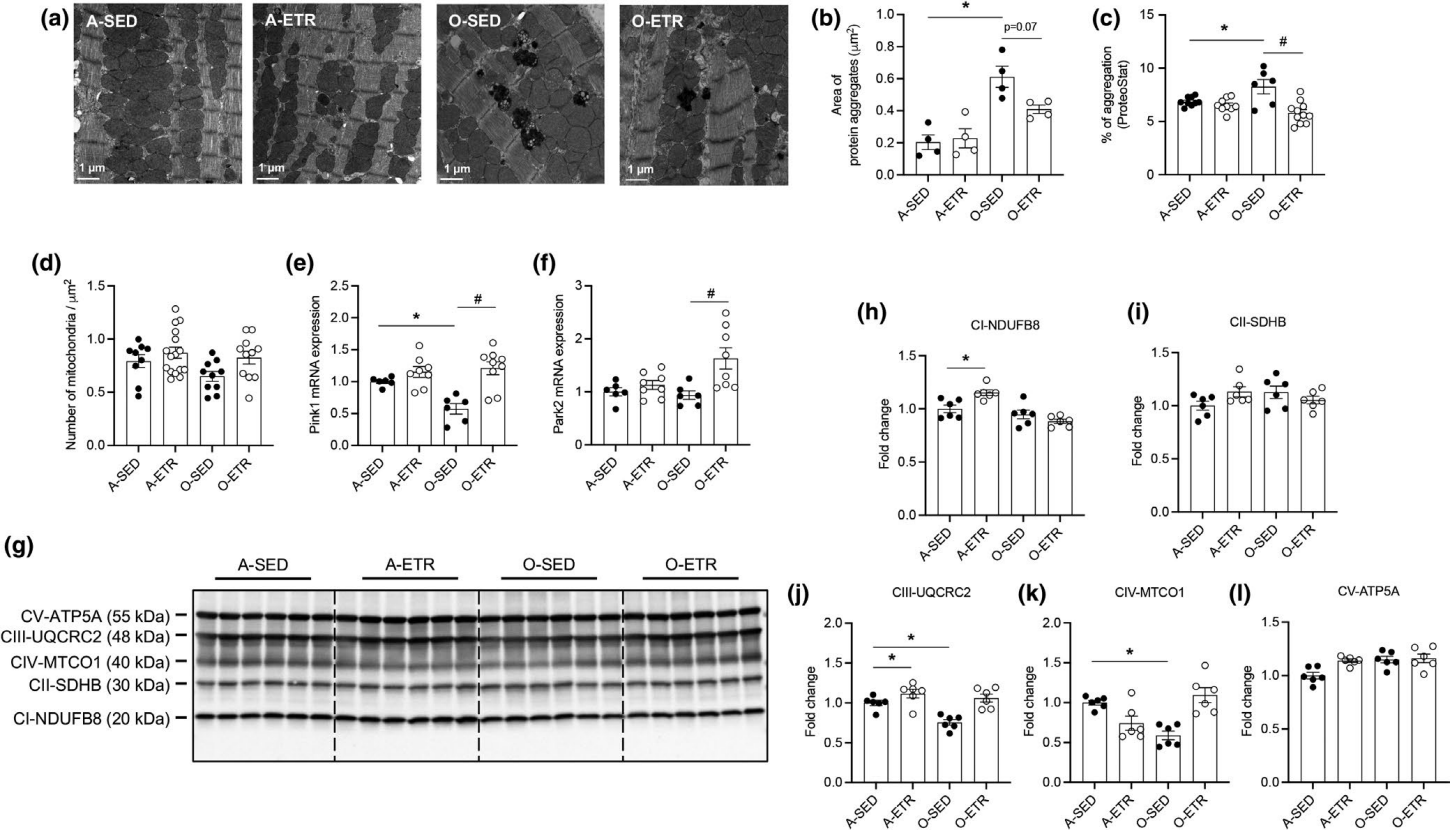
Late‐in‐life exercise training attenuates protein aggregate accrual, and improves indexes of mitophagy and mitochondrial quality in mouse hearts. Adult (A, 5 mo) and older (O, 21 mo) mice did (ETR) or did not (SED) complete 12 wk of treadmill running. At least 24 h following the last exercise bout, hearts were obtained from A‐SED, A‐ETR (8 mo), and O‐SED, O‐ETR (24 mo) mice. Protein aggregates (a–c) and mitochondrial number (d; electron microscopy, EM), PTEN‐induced kinase 1 (Pink1; e) and Parkinson protein 2 E3 ubiquitin protein ligase isoform 3 (Park2, f; qPCR), and Complex I‐V protein expression (g–l; immunoblotting) were assessed. (a) Representative EM images (2700×) from each group. Aging‐associated protein aggregation (a, b) was attenuated (*p *= 0.07) by late‐in‐life exercise training. Alternative procedures (ProteoStat assay kit; c) substantiate results shown in (b). Mitochondrial number did not differ among groups (d). The age‐associated reduction in Pink1 mRNA was restored by late‐in‐life exercise training (e), whereas training improved Park2 mRNA in older mice (f). The age‐associated reduction in UQCRC2 (j, complex III) and MTCO1 (k, complex IV) was restored by exercise training. Aging did not impact NDUFB8 (h; complex I), SDHB (i, complex II), or ATP5A (l, complex V). For (b), *n* = 4 mice per group, *n* = 8–16 fields of view, data are expressed as area of protein aggregation (µm^2^); (c), *n* = 6–11, data are expressed as % of protein aggregation; (d), *n* = 9–16, data expressed as number of mitochondria per area (µm^2^); (e, f), *n* = 6–8 mice; (g–l), *n* = 6 mice; (b–k), *p < 0.05 vs. A‐SED; #*p *< 0.05 vs. O‐SED

Electron microscopy images indicate aging‐associated cardiac protein aggregation is normalized by exercise training (Figure 6a, b), whereas mitochondrial number did not change (Figure 6a, d). Protein aggregation results were substantiated using an alternative procedure (i.e., Proteostat; Figure 6c). Age‐associated reductions in PTEN‐induced kinase 1 (Pink1) gene expression were normalized by exercise training (Figure 6e), whereas 3 months of treadmill running improved Parkin RBR E3 ubiquitin protein ligase (Park2) in older mice (Figure 6f). Complex I, II, and V of the electron transport chain were not different in adult vs. older mice, but age‐associated reductions in Complex III (Figure 6g, j) and IV (Figure 6g, k) protein expression were restored by exercise‐training. Ponceau S (Figure S15) was used as a loading control for results shown in Figure 6g‐l. Taken together, these findings indicate protein aggregate removal and mitochondrial quality is improved in mouse hearts by late‐in‐life exercise training.

## 3 DISCUSSION

Our primary aim was to test the hypothesis that late‐in‐life exercise training rejuvenates indexes of cardiac autophagy, improves clearance of ubiquitinated proteins, and re‐establishes cardiac function. First, we substantiated earlier findings that repressed autophagic flux in the heart of older vs. adult mice exists, and that this is associated with protein aggregate accrual, oxidative stress, and cardiac dysfunction. Next, we demonstrated for the first time that a physiological intervention, that is, progressive resistance treadmill running, improves autophagic flux, protein clearance, redox balance, mitochondrial quality, and cardiac function, in hearts from older mice. These data indicate positive crosstalk exists between regular physical activity and cardiac autophagy in the context of primary aging.

### 3.1 Autophagic flux is depressed in hearts from older mice

Repressed autophagy is observed in a wide variety of conditions associated with aging, including neurodegenerative diseases, normal brain aging, osteoarthritis, insulin resistance, atherosclerosis, macular degeneration, suppressed hepatic proteolysis, heart failure, and endothelial cell dysfunction (Bharath et al., 2017; Campos et al., 2017; Park et al., 2019; Pires et al., 2017; Rubinsztein et al., 2011). A close examination of the literature reveals that the impact of aging on cardiac autophagy in pre‐clinical murine models is not congruent. Most studies investigating this issue have assessed steady‐state autophagy by quantifying protein expression of LC3‐II and/or p62. The membrane‐bound lipidated form of cytosolic LC3‐I, that is, LC3‐II accumulates as the phagophore membrane is formed and extended during the process of autophagy. Atg3 is the phosphatidylethanolamine‐transferase that performs the final lipid conjugation modification of LC3 required for completing the conversion of cytosolic LC3‐I to membrane‐bound LC3‐II (Mizushima, 2007). The adaptor protein p62, which tethers targeted cargo destined to become engulfed in the autophagosome, is degraded as autophagy proceeds.

A variety of studies indicate p62 accumulates in hearts from aged vs. adult mice (Li et al., 2020; Liang et al., 2020; Ren et al., 2017; Wang et al., 2018; Wu et al., 2016; Zhang et al., 2017), and translational relevance of these findings to older humans was recently reported (Li et al., 2020). Results concerning LC3‐II are less clear. With regard to C57BL/6J mice: (i) LC3II:GAPDH (Liang et al., 2020; Taneike et al., 2010) and LC3‐II:LC3‐I (Ren et al., 2017) declined in hearts from ~26 months vs. ~4 months animals; (ii) LC3‐II/LC3‐I increased in 18 months vs. 2 months mice; (Boyle et al., 2011) and (iii) LC3‐II/LC3‐I was not different between ~23 months and ~4 months animals (Li et al., 2020; Wu et al., 2016). We observed increased LC3‐I:GAPDH, LC3‐II:GAPDH, and p62:GAPDH in older vs. adult mice from two independent cohorts treated identically (Figures 1, 3). Because Atg3 mRNA and protein expression was lower in older vs. adult mice (Figures S1, S2, S6), elevated LC3‐I:GAPDH observed in older animals might result from an inability to perform the lipid conjugation step whereby cytosolic LC3‐I is converted to membrane‐bound LC3‐II. With regard to LC3‐II and p62 accrual observed in hearts from older vs. adult mice, this might be secondary to a defect that exists later in the process of autophagy and we tested this. Separate cohorts of adult and old mice were treated with the autophagosome‐lysosome fusion inhibitor CQ to assess autophagic flux (Gottlieb et al., 2015; Klionsky et al., 2021; Pires et al., 2017). This approach has been used in the context of cardiac aging on two occasions. Wu et al. treated C57BL/6J mice with the vacuolar H^+^‐ATPase inhibitor bafilomycin (0.3 mg/kg IP × 7 days), which impairs lysosomal acidification, blocks autophagosome‐lysosome fusion, and thereby prevents degradation of autophagolysosomes. Compared to mice that were administered a vehicle‐control, bafilomycin increased LC3‐II: LC3‐I and p62 protein expression in cardiac lysates from 4 but not 22‐mo‐old mice (Wu et al., 2016). Using a different species and experimental setting, Ma et al. reported that cardiomyocytes isolated from hearts of 4 months rats displayed greater LC3 puncta and p62 expression after treatment with bafilomycin (100 nM × ~4 h) compared to results obtained from ~24 months rats (Ma et al., 2017). Both studies concluded that constitutive autophagosome formation is robust in hearts from adult but not older mice and our results after 4 h (Figure 1) and 48 h (Figure 4) CQ administration are supportive. Specifically, CQ increased LC3‐II:GAPDH and p62:GAPDH in hearts from adult but not older mice (Figures 1, 4). These data substantiate that autophagosome clearance capacity is compromised in aged mouse hearts.

### 3.2 Repressed cardiac autophagic flux associates with proteotoxicity, oxidative stress, impaired mitochondrial quality, and contractile dysfunction

Strong rationale exists that repressed autophagosome formation contributes importantly to accelerated cardiac aging. In a loss of autophagy approach, adult mice with cardiac‐selective Atg5 deletion (Taneike et al., 2010), and cardiac‐specific mTOR activation via miR‐199a overexpression (Li et al., 2017) or tuberous sclerosis complex 1 and 2 depletion (Taneike et al., 2016), exhibit important characteristics of cardiac aging, that is, protein aggregate accrual, interstitial fibrosis, LV hypertrophy, oxidative stress, and/or cardiac dysfunction. In addition to compromised autophagic flux, old vs. adult mice in the present study displayed each of these features of cardiac aging. Highlighting an association between repressed cardiac autophagy and cardiac dysfunction, elevated cardiac p62 protein expression correlated significantly with a well‐accepted estimate of overall LV dysfunction, that is, the MPI (Figure 2m) (Goroshi & Chand, 2016). Using a gain of autophagy procedure, mice with cardiac‐selective Atg7 overexpression (*Atg7* transgenic mice) were crossed with *CryAB^R120G^* mice, a model of desmin‐related cardiomyopathy that exhibits impaired autophagic flux together with the accumulation of preamyloid oligomer (PAO), a toxic component in many of the protein misfolding based neurodegenerative diseases (Maloyan et al., 2007; Pattison et al., 2011). As anticipated, autophagic flux was greater, and accrual of cytotoxic proteins, impaired cardiac performance, and early mortality was less severe, in *CryAB^R120G^* × *Atg7* transgenic mice vs. *CryAB^R120G^* animals (Bhuiyan et al., 2013). Based on previous results using loss of autophagy and gain of autophagy approaches involving the heart, it is not unreasonable to suggest that impaired autophagic flux (Figures 1, 4) contributed importantly to the accrual of ubiquitinated proteins and elevated lipid peroxidation (Figures 1, 3), protein aggregate accumulation (Figure 6), increased fibrosis (Figure S2), and systolic and diastolic dysfunction (Figure 2, Figure S3) exhibited by older vs. adult hearts in our study. While precise mechanisms responsible for the age‐associated reduction in Atg3 (Figures S1, S2, S6) in cardiomyocytes have not been reported, evidence exists that oxidative stress inhibits Atg3 enzyme function in HEK 293 cells (Frudd et al., 2018) and Atg3 protein expression in mouse brain endothelial cells (Kamat et al., 2015). Because autophagy is an important driver of mitochondrial clearance, stalled mitophagy in aged hearts could precipitate reactive oxygen species generation from dysregulated mitochondria, and our results concerning repressed Pink1, Complex III, and Complex IV in hearts from older vs. adult mice support this notion (Figure 6).

### 3.3 Late‐in‐life interventions that activate autophagy associate with attenuated cardiac dysfunction

Genetic manipulations (e.g., Atg7 overexpression) cannot be used clinically to upregulate autophagy in conditions associated with cardiac proteotoxicity at present. However, benefits from inducing this protein degradation pathway late‐in‐life via nutraceutical (e.g., spermidine), lifestyle (e.g., caloric restriction), and pharmacological (e.g., rapamycin) maneuvers have been demonstrated (Eisenberg et al., 2016; Flynn et al., 2013; Sheng et al., 2017). While each of these autophagy‐boosting approaches attenuated age‐associated cardiac dysfunction (Eisenberg et al., 2016; Flynn et al., 2013; Sheng et al., 2017), it is unknown if functional benefits associated positively with improved autophagic flux and protein clearance in the heart because neither of these endpoints was assessed.

A lifestyle intervention with potential to improve autophagy, clear damaged proteins, and beneficially influence the aging‐associated decline in cardiac function is dynamic exercise. He et al. first showed in mice that 30–80‐min treadmill running increases LC3‐GFP puncta, LC3‐II:LC3‐I, and p62 degradation in the heart (He et al., 2012). Beta cell lymphoma/leukemia 2 (Bcl‐2) is an anti‐apoptotic and anti‐autophagy protein that inhibits autophagy by directly interacting with beclin 1 at the endoplasmic reticulum. The authors reported that the Bcl‐2–beclin‐1 complex dissociates in response to treadmill running, and this finding was confirmed by Bhuiyan et al. in mice that completed an acute bout of voluntary wheel running (VWR) (Bhuiyan et al., 2013). Because long‐term VWR decreased the amyloid load in mice with neurodegenerative disorders, for example, Alzheimer's disease (Lazarov et al., 2005) the Robbins laboratory group sought to determine whether this form of “environmental enrichment” initiates cardiac autophagy to an extent that improves cardiac proteostasis. Providing strong proof of concept, the authors reported a 47% reduction in cardiac PAO accumulation in *CryAB^R120G^* mice that completed 6 months of VWR vs. *CryAB^R120G^* animals that did not train, but indexes of autophagy were not assessed (Maloyan et al., 2007). The same investigative team later demonstrated that VWR increased mRNA expression of Atg4, Atg5, and Wipi1 in hearts from *CryAB^R120G^* mice vs. untrained mice, but neither autophagic flux nor protein aggregate accrual were assessed (Bhuiyan et al., 2013). In the latter study, it is extremely interesting to note that functional endpoints assessed via echocardiography (LVIDs, LVIDd, and EF) appear identical between *CryAB^R120G^* × *Atg7* transgenic mice and *CryAB^R120G^* mice that completed VWR, suggesting that exercise training conferred benefits similar to genetic autophagy activation and vice versa.

### 3.4 Late‐in‐life exercise training improves cardiac autophagic flux that associates positively with cardiac function

The interesting findings from interventions involving nutraceuticals, pharmaceuticals, and lifestyle alterations in older mice inspired us to test whether late‐in‐life exercise training induces cardiac autophagy to an extent that improves proteostasis and lessens cardiac dysfunction. As anticipated, the intensity, frequency, and duration of “forced” exercise training produced functional and biochemical evidence of efficacy (Figure S5). In support of our hypothesis, age‐associated dysregulation of LC3‐II:GAPDH and p62:GAPDH improved in hearts from old‐ETR vs. old‐SED mice (Figures 3, 4). Importantly, 4 and 48 h CQ‐induced LC3‐II:GAPDH and p62:GAPDH accumulation occurred in hearts from old‐ETR but not old‐SED mice (Figure 4, Figures S8, S9). Collectively, these results indicate that 3‐month treadmill running improves steady‐state autophagy and autophagic flux in hearts from older mice, and these observations are concurrent with (i) heightened clearance of ubiquitinated proteins (Figure 3); (ii) reduced lipid peroxidation (Figure 3); and (iii) attenuated protein aggregation (Figure 6). It is not unreasonable to suggest that elevated Atg3 mRNA and protein expression in hearts from old‐ETR vs. old‐SED mice (Figures S6, S7, S9) enabled phagophore maturation to facilitate autophagosome formation. While the mechanism(s) responsible for elevated Atg3 in old‐ETR vs. old‐SED mice is unclear and to our knowledge has not been reported, improved redox balance (Figure 3) displayed by hearts from old‐ETR mice might be responsible, based on results from studies using other cell types (Frudd et al., 2018; Kamat et al., 2015). In our investigation, elevated Pink1 and Park2 mRNA in hearts from old‐ETR vs. old‐SED mice represent a greater ability to clear ROS generating mitochondria to an extent that normalizes mitochondrial quality (Figure 6). We realize that associations are presented and cause and effect relationships remain to be identified.

Three months of treadmill training did not prevent aging‐associated cardiac fibrosis (Figure S11a–d). However, multiple indexes of systolic performance, together with a doppler‐derived measure of overall LV function that incorporates the time intervals of mitral valve inflow and aortic valve outflow (i.e., MPI) (Goroshi & Chand, 2016), improved in trained vs. untrained older mice (Figure 5). Highlighting the association between training‐induced benefits concerning cardiac autophagy (i.e., reduced cardiac p62 protein expression) and LV function (i.e., lower MPI), a significant correlation existed between these two endpoints in older trained mice (Figure 5l). On balance, our data indicate that late‐in‐life exercise training rejuvenates autophagic flux that associates positively with (i) clearance of dysregulated mitochondria, ubiquitinated proteins, and protein aggregates; (ii) improved redox balance; and (iii) attenuated cardiac dysfunction.

We observed a strong association between the age‐related accrual of p62:GAPDH (repressed autophagy) and the increase (i.e., worsening) of MPI (Figure 2m); and the training‐induced reduction in p62:GAPDH (improved autophagy) and the decrease (i.e., improvement) of MPI (Figure 5l). While these findings indicate training‐induced elevations in autophagic flux associate positively with preserved cardiac function in the context of primary aging, evidence exists that an 8‐week exercise program preserves autophagic flux in adult rats in the setting of a common age‐related pathology, for example, heart failure (Campos et al., 2017). Specifically, 12 weeks following myocardial infarction‐induced heart failure via left anterior descending coronary artery ligation, indexes of cardiac autophagic flux and cardiac function were improved in rats that completed treadmill running from weeks 4–12 vs. those that did not train. At present, it is unknown whether late‐in‐life exercise training rejuvenates autophagic flux to an extent that improves tolerance to infarction‐induced heart failure, but these studies are ongoing in our laboratory. Two substrains of C57BL/6 mice were used in the present study, that is, C57BL/6J for adult mice (Jackson Laboratories) and C57BL/6N for older mice (NIA rodent colony). While both substrains share the same core genetic background, nuances do exist which could influence the phenotypes we describe, and this limitation should be considered when integrating our findings into what is known currently.

## 4 EXPERIMENTAL PROCEDURES

### 4.1 Animals and housing

Male C57BL/6J mice were obtained from the Jackson Laboratories at 4 months of age, and C57BL/6N animals were obtained from the National Institute on Aging rodent colony at 18 months of age. All mice were housed 4‐per cage under controlled temperature (22°C) and light (12:12‐h light‐dark cycle) conditions, and were provided with food (Diet #2920, Teklad Diets, Madison, WI) and water ad libitum. Animals were handled according to Institutional approved procedures documented in protocol number 19‐07010 JDS.

### 4.2 Cardiac autophagy

Steady‐state (i.e., basal) cardiac autophagy and autophagosome formation (i.e., autophagic flux) were measured in 24‐month‐ and 8‐month‐old mice (Figure 1a). Body composition was assessed using time‐domain‐nuclear magnetic resonance (TD‐NMR; Bruker minispec, Bruker Biospin Corporation) (Bharath et al., 2015, 2017). Twenty‐four h later, chloroquine (CQ; 75 mg/g lean body mass) or vehicle‐control (phosphate‐buffered saline; PBS) (Gottlieb et al., 2015; Pires et al., 2017) was administered IP to separate cohorts of older and adult mice. Four hours later (Klionsky et al., 2021), mice were anesthetized using an inhaled mixture of 2%–4% isoflurane combined with 100% oxygen, and hearts were obtained to assess mRNA and protein indexes of autophagy (Bharath et al., 2017; Pires et al., 2017), ubiquitinated proteins, and 4‐hydroxy‐2‐nonenal (4‐HNE), an α, β‐unsaturated hydroxyalkenal that is an estimate of lipid peroxidation. Protein isolation and immunoblotting analyses were performed as we described (Bharath et al., 2017; Bharath et al., 2015; Symons et al., 2011; Zhang et al., 2012). This is referred to in Figures 1, S1, S8, and S9 as the 4h CQ protocol. In a different cohort of adult and older mice that did or did not train, CQ was administered IP 48 h (30 mg/kg), 24 h (30 mg/kg), and 4h (50 mg/kg) prior to tissue collection (Figures 4, S10) (Ju et al., 2010). This is referred to in Figures 4 and S10 as the 48 h CQ protocol. For all protocols, hearts were obtained at the same time of day. A list of primers (Table S2), antibodies (Table S3), and other materials (Table S4) used in this study is provided.

### 4.3 Cardiac function

Separate cohorts of adult and older mice were anesthetized lightly with 1%–3% inhaled isoflurane combined with 100% oxygen, and transthoracic echocardiography was completed to assess indexes of systolic and diastolic function (Pires et al., 2017; Symons et al., 2011). Mice were recovered from anesthesia following the echocardiography measurements. These procedures were initiated at the same time of day.

### 4.4 Histology, morphology, mRNA gene expression

Twenty‐four to 48 h after assessing cardiac function, mice were anesthetized using 2%–4% inhaled isoflurane combined with 100% oxygen and hearts were obtained to assess protein indexes of autophagy, histology, morphology, and mRNA expression of profibrotic and antioxidant genes (Bharath et al., 2017; Bharath et al., 2015; Symons et al., 2011; Zhang et al., 2012).

### 4.5 Exercise training

Body composition was assessed using TD‐NMR in sym5‐month‐ and 21‐month‐old mice. Twenty‐four to 48 h later, mice were familiarized with walking/running on a motorized treadmill (Columbus Instruments). On day 4, a workload capacity evaluation test was completed on each mouse. Total workload was calculated as [body weight (kg) × total running time (min) × final running speed (m/min) × treadmill grade (25%)] (Symons et al., 2000). After all mice finished the workload capacity evaluation, they were separated randomly into groups that did not (adult‐SED and old‐SED) or did (adult‐ETR and old‐ETR) complete a 3‐month progressive resistance treadmill running program. Exercise‐training bouts were completed between 0600 and 1000 each day. After 3 months, body composition, exercise tolerance (maximal workload capacity), and cardiac function were assessed. Each evaluation was separated by 24 h and completed at the same time of day. Twenty‐four hours after measuring cardiac function, all mice were anesthetized as described, and the heart was excised to assess protein indexes of autophagy, histology, morphology, mRNA expression of profibrotic (described earlier) and mitophagy‐related genes, protein aggregate accumulation, and protein expression of Complex I‐V of the electron transport chain. Soleus muscle was dissected free from both hindlimbs to assess CS enzyme activity (Sigma‐Aldrich) (Symons et al., 2000).

### 4.6 Mitochondrial number and quality

Mitochondrial number was assessed using electron microscopy, mitophagy‐related gene expression was assessed using qPCR, and Complex I‐V protein expression of the electron transport chain was assessed by immunoblotting. Details are provided in online “Experimental procedures.”

### 4.7 Cardiac protein aggregation

After determining cardiac protein concentrations (Pierce BCA Protein Assay; ThermoFisher), protein aggregate accrual in the heart was measured using a commercially available kit (Proteostat; Enzo Life Sciences) (Laor et al., 2019). Electron microscopy was used as an additional approach to estimate cardiac protein aggregates (DiMemmo et al., 2017). Details are provided in online “Experimental procedures.”

### 4.8 Statistical analyses

Data are presented as mean ± standard of error of the mean. Significance was accepted when *p* < 0.05. To determine normality of the distribution for each data set, GraphPad Prism software was used. An unpaired t test (e.g., EF in adult vs. old mice) was used, as appropriate, to compare two mean values. Comparison among four means was completed using a one‐way ANOVA (e.g., cardiac p62:GAPDH among old‐SED‐VEH, old‐SED‐CQ, old‐ETR‐VEH, and old‐ETR‐CQ). In cases when a significant main effect was obtained, a Tukey post hoc test was used to determine the location of the differences.

## CONFLICT OF INTEREST

None of the authors has any conflicts of interest to disclose.

## AUTHOR CONTRIBUTIONS

JMC, SB, and JDS designed the study. JMC, KL, CR, LT, MH, MSLCM, KMP, MF, MB, RG, SKP, and JDS performed experiments and/or trained the mice. KW and KC performed echocardiography at the UU Small Animal Ultrasound Facility. JMC, SB, MH, and JDS analyzed the data. JMC, SB, and JDS wrote the manuscript.

## Supporting information

Figure S1

Figure S2

Figure S3

Figure S4

Figure S5

Figure S6

Figure S7

Figure S8

Figure S9

Figure S10

Figure S11

Figure S12

Figure S13

Figure S14

Figure S15

Supplementary Material

## Data Availability

The raw data supporting the conclusions of this manuscript will be made available by the authors, without undue reservation, to any qualified researcher.

## References

[acel13467-bib-0001] Benjamin, E. J. , Muntner, P. , Alonso, A. , Bittencourt, M. S. , Callaway, C. W. , Carson, A. P. , Chamberlain, A. M. , Chang, A. R. , Cheng, S. , Das, S. R. , Delling, F. N. , Djousse, L. , Elkind, M. S. V. , Ferguson, J. F. , Fornage, M. , Jordan, L. C. , Khan, S. S. , Kissela, B. M. , Knutson, K. L. , … Virani, S. S. (2019). Heart disease and stroke statistics‐2019 update: A report from the American Heart Association. Circulation, 139(10), e56–e528. 10.1161/CIR.0000000000000659 30700139

[acel13467-bib-0002] Bharath, L. P. , Cho, J. M. , Park, S.‐K. , Ruan, T. , Li, Y. , Mueller, R. , Bean, T. , Reese, V. , Richardson, R. S. , Cai, J. , Sargsyan, A. , Pires, K. , Anandh Babu, P. V. , Boudina, S. , Graham, T. E. , Symons, J. D. (2017). Endothelial Cell Autophagy maintains shear stress‐induced nitric oxide generation via glycolysis‐dependent purinergic signaling to endothelial nitric oxide synthase. Arteriosclerosis, Thrombosis, and Vascular Biology, 37(9), 1646–1656. 10.1161/ATVBAHA.117.309510 PMC5693355 28684613

[acel13467-bib-0003] Bharath, L. P. , Ruan, T. , Li, Y. , Ravindran, A. , Wan, X. , Nhan, J. K. , Walker, M. L. , Deeter, L. , Goodrich, R. , Johnson, E. , Munday, D. , Mueller, R. , Kunz, D. , Jones, D. , Reese, V. , Summers, S. A. , Babu, P. V. A. , Holland, W. L. , Zhang, Q.‐J. , … Symons, J. D. (2015). Ceramide‐initiated protein phosphatase 2A activation contributes to arterial dysfunction in vivo. Diabetes, 64(11), 3914–3926. 10.2337/db15-0244 26253611 PMC4613970

[acel13467-bib-0004] Bhuiyan, M. S. , Pattison, J. S. , Osinska, H. , James, J. , Gulick, J. , McLendon, P. M. , Hill, J. A. , Sadoshima, J. , Robbins, J. (2013). Enhanced autophagy ameliorates cardiac proteinopathy. Journal of Clinical Investigation, 123(12), 5284–5297. 10.1172/JCI70877 PMC3859422 24177425

[acel13467-bib-0005] Boyle, A. J. , Shih, H. , Hwang, J. , Ye, J. , Lee, B. , Zhang, Y. , Kwon, D. , Jun, K. , Zheng, D. , Sievers, R. , Angeli, F. , Yeghiazarians, Y. , Lee, R. (2011). Cardiomyopathy of aging in the mammalian heart is characterized by myocardial hypertrophy, fibrosis and a predisposition towards cardiomyocyte apoptosis and autophagy. Experimental Gerontology, 46(7), 549–559. S0531‐5565(11)00060‐X [pii]; 10.1016/j.exger.2011.02.010 21377520 PMC3104129

[acel13467-bib-0006] Campos, J. C. , Queliconi, B. B. , Bozi, L. H. M. , Bechara, L. R. G. , Dourado, P. M. M. , Andres, A. M. , Jannig, P. R. , Gomes, K. M. S. , Zambelli, V. O. , Rocha‐Resende, C. , Guatimosim, S. , Brum, P. C. , Mochly‐Rosen, D. , Gottlieb, R. A. , Kowaltowski, A. J. , Ferreira, J. C. B. (2017). Exercise reestablishes autophagic flux and mitochondrial quality control in heart failure. Autophagy, 13(8), 1304–1317. 10.1080/15548627.2017.1325062 28598232 PMC5584854

[acel13467-bib-0007] Cuervo, A. M. , & Dice, J. F. (2000). Age‐related decline in chaperone‐mediated autophagy. Journal of Biological Chemistry, 275(40), 31505–31513. 10.1074/jbc.M002102200[doi];M002102200 [pii] 10806201

[acel13467-bib-0008] Dai, D. F. , & Rabinovitch, P. S. (2009). Cardiac aging in mice and humans: the role of mitochondrial oxidative stress. Trends in Cardiovascular Medicine, 19(7), 213–220. S1050‐1738(09)00182‐0 [pii]; 10.1016/j.tcm.2009.12.004[doi] 20382344 PMC2858758

[acel13467-bib-0009] Dai, D. F. , Rabinovitch, P. S. , & Ungvari, Z. (2012). Mitochondria and cardiovascular aging. Circulation Research, 110(8), 1109–1124. 110/8/1109 [pii]; 10.1161/CIRCRESAHA.111.246140[doi] 22499901 PMC3867977

[acel13467-bib-0010] Dai, D. F. , Santana, L. F. , Vermulst, M. , Tomazela, D. M. , Emond, M. J. , MacCoss, M. J. , Gollahon, K. , Martin, G. M. , Loeb, L. A. , Ladiges, W. C. , Rabinovitch, P. S. (2009). Overexpression of catalase targeted to mitochondria attenuates murine cardiac aging. Circulation, 119(21), 2789–2797. CIRCULATIONAHA.108.822403 [pii]; 10.1161/CIRCULATIONAHA.108.822403[doi] 19451351 PMC2858759

[acel13467-bib-0011] DiMemmo, L. M. , Cameron Varano, A. , Haulenbeek, J. , Liang, Y. , Patel, K. , Dukes, M. J. , Zheng, S. , Hubert, M. , Piccoli, S. P. , Kelly, D. F. (2017). Real‐time observation of protein aggregates in pharmaceutical formulations using liquid cell electron microscopy. Lab on a Chip, 17(2), 315–322. 10.1039/c6lc01160h 27934977 PMC5507349

[acel13467-bib-0012] Eisenberg, T. , Abdellatif, M. , Schroeder, S. , Primessnig, U. , Stekovic, S. , Pendl, T. , Harger, A. , Schipke, J. , Zimmermann, A. , Schmidt, A. , Tong, M. , Ruckenstuhl, C. , Dammbrueck, C. , Gross, A. S. , Herbst, V. , Magnes, C. , Trausinger, G. , Narath, S. , Meinitzer, A. , … Madeo, F. (2016). Cardioprotection and lifespan extension by the natural polyamine spermidine. Nature Medicine, 22(12), 1428–1438. nm.4222 [pii]; 10.1038/nm.4222[doi] PMC5806691 27841876

[acel13467-bib-0013] Flynn, J. M. , O'Leary, M. N. , Zambataro, C. A. , Academia, E. C. , Presley, M. P. , Garrett, B. J. , Zykovich, A. , Mooney, S. D. , Strong, R. , Rosen, C. J. , Kapahi, P. , Nelson, M. D. , Kennedy, B. K. , Melov, S. (2013). Late‐life rapamycin treatment reverses age‐related heart dysfunction. Aging Cell, 12(5), 851–862. 10.1111/acel.12109[doi] 23734717 PMC4098908

[acel13467-bib-0014] Frudd, K. , Burgoyne, T. , & Burgoyne, J. R. (2018). Oxidation of Atg3 and Atg7 mediates inhibition of autophagy. Nature Communications, 9(1), 95. 10.1038/s41467-017-02352-z PMC5758830 29311554

[acel13467-bib-0015] Goroshi, M. , & Chand, D. (2016). Myocardial Performance Index (Tei Index): A simple tool to identify cardiac dysfunction in patients with diabetes mellitus. Indian Heart Journal, 68(1), 83–87. S0019‐4832(15)00248‐5 [pii]; 10.1016/j.ihj.2015.06.022[doi] 26896273 PMC4759491

[acel13467-bib-0016] Gottlieb, R. A. , Andres, A. M. , Sin, J. , & Taylor, D. P. (2015). Untangling autophagy measurements: all fluxed up. Circulation Research, 116(3), 504–514. CIRCRESAHA.116.303787 [pii]; 10.1161/CIRCRESAHA.116.303787[doi] 25634973 PMC4313387

[acel13467-bib-0017] He, C. , Bassik, M. C. , Moresi, V. , Sun, K. , Wei, Y. , Zou, Z. , An, Z. , Loh, J. , Fisher, J. , Sun, Q. , Korsmeyer, S. , Packer, M. , May, H. I. , Hill, J. A. , Virgin, H. W. , Gilpin, C. , Xiao, G. , Bassel‐Duby, R. , & Scherer, P. E. , Levine, B. (2012). Exercise‐induced BCL2‐regulated autophagy is required for muscle glucose homeostasis. Nature, 481(7382), 511–515. nature10758 [pii]; 10.1038/nature10758[doi] 22258505 PMC3518436

[acel13467-bib-0018] Ju, J. S. , Varadhachary, A. S. , Miller, S. E. , & Weihl, C. C. (2010). Quantitation of "autophagic flux" in mature skeletal muscle. Autophagy, 6(7), 929–935. 10.4161/auto.6.7.12785 20657169 PMC3039739

[acel13467-bib-0019] Kamat, P. K. , Kalani, A. , Tyagi, S. C. , & Tyagi, N. (2015). Hydrogen sulfide epigenetically attenuates homocysteine‐induced mitochondrial toxicity mediated through NMDA receptor in mouse brain endothelial (bEnd3) cells. Journal of Cellular Physiology, 230(2), 378–394. 10.1002/jcp.24722 25056869 PMC4305357

[acel13467-bib-0020] Klionsky, D. J. , Abdel‐Aziz, A. K. , Abdelfatah, S. , Abdellatif, M. , Abdoli, A. , Abel, S. , Abeliovich, H. , Abildgaard, M. H. , Abudu, Y. P. , Acevedo‐Arozena, A. , Adamopoulos, I. E. , Adeli, K. , Adolph, T. E. , Adornetto, A. , Aflaki, E. , Agam, G. , Agarwal, A. , Aggarwal, B. B. , & Agnello, M. … Tong, C. K. (2021). Guidelines for the use and interpretation of assays for monitoring autophagy (4th edition)(1). Autophagy, 17(1), 1–382. 10.1080/15548627.2020.1797280 33634751 PMC7996087

[acel13467-bib-0021] Koga, H. , Kaushik, S. , & Cuervo, A. M. (2011). Protein homeostasis and aging: The importance of exquisite quality control. Ageing Research Reviews, 10(2), 205–215. S1568‐1637(10)00005‐X [pii]; 10.1016/j.arr.2010.02.001[doi] 20152936 PMC2888802

[acel13467-bib-0022] Laor, D. , Sade, D. , Shaham‐Niv, S. , Zaguri, D. , Gartner, M. , Basavalingappa, V. , Raveh, A. , Pichinuk, E. , Engel, H. , Iwasaki, K. , Yamamoto, T. , Noothalapati, H. , Gazit, E. (2019). Fibril formation and therapeutic targeting of amyloid‐like structures in a yeast model of adenine accumulation. Nature Communications, 10(1), 62. 10.1038/s41467-018-07966-5 PMC6325136 30622276

[acel13467-bib-0023] Lazarov, O. , Robinson, J. , Tang, Y. P. , Hairston, I. S. , Korade‐Mirnics, Z. , Lee, V. M. , Hersh, L. B. , Sapolsky, R. M. , Mirnics, K. , Sisodia, S. S. (2005). Environmental enrichment reduces Abeta levels and amyloid deposition in transgenic mice. Cell, 120(5), 701–713. 10.1016/j.cell.2005.01.015 15766532

[acel13467-bib-0024] Li, C. , Mu, N. , Gu, C. , Liu, M. , Yang, Z. , Yin, Y. , Chen, M. , Wang, Y. , Han, Y. , Yu, L. U. , Ma, H. (2020). Metformin mediates cardioprotection against aging‐induced ischemic necroptosis. Aging Cell, 19(2), e13096. 10.1111/acel.13096 31944526 PMC6996959

[acel13467-bib-0025] Li, C. , Yu, L. , Xue, H. , Yang, Z. , Yin, Y. , Zhang, B. , Chen, M. , Ma, H. (2017). Nuclear AMPK regulated CARM1 stabilization impacts autophagy in aged heart. Biochemical and Biophysical Research Communications, 486(2), 398–405. S0006‐291X(17)30511‐9 [pii]; 10.1016/j.bbrc.2017.03.053[doi] 28315332

[acel13467-bib-0026] Liang, W. , Moyzis, A. G. , Lampert, M. A. , Diao, R. Y. , Najor, R. H. , & Gustafsson, A. B. (2020). Aging is associated with a decline in Atg9b‐mediated autophagosome formation and appearance of enlarged mitochondria in the heart. Aging Cell, 19(8), e13187. 10.1111/acel.13187 32627317 PMC7431832

[acel13467-bib-0027] Ma, L. , Zhu, J. , Gao, Q. , Rebecchi, M. J. , Wang, Q. , & Liu, L. (2017). Restoring pharmacologic preconditioning in the aging heart: role of mitophagy/autophagy. Journals of Gerontology. Series A, Biological Sciences and Medical Sciences, 72(4), 489–498. glw168. [pii]; 10.1093/gerona/glw168[doi] 27565512

[acel13467-bib-0028] Maloyan, A. , Gulick, J. , Glabe, C. G. , Kayed, R. , & Robbins, J. (2007). Exercise reverses preamyloid oligomer and prolongs survival in alphaB‐crystallin‐based desmin‐related cardiomyopathy. Proceedings of the National Academy of Sciences of the United States of America, 104(14), 5995–6000. 0609202104. [pii]; 10.1073/pnas.0609202104[doi] 17389375 PMC1851605

[acel13467-bib-0029] Mizushima, N. (2007). Autophagy: process and function. Genes & Development, 21(22), 2861–2873. 21/22/2861 [pii]; 10.1101/gad.1599207[doi] 18006683

[acel13467-bib-0030] Park, S. K. , La Salle, D. T. , Cerbie, J. , Cho, J. M. , Bledsoe, A. , Nelson, A. , Morgan, D. E. , Richardson, R. S. , Shiu, Y.‐T. , Boudina, S. , Trinity, J. D. , Symons, J. D. (2019). Elevated arterial shear rate increases indexes of endothelial cell autophagy and nitric oxide synthase activation in humans. American Journal of Physiology‐Heart and Circulatory Physiology, 316(1), H106–H112. 10.1152/ajpheart.00561.2018 30412436 PMC6734082

[acel13467-bib-0031] Pattison, J. S. , Osinska, H. , & Robbins, J. (2011). Atg7 induces basal autophagy and rescues autophagic deficiency in CryABR120G cardiomyocytes. Circulation Research, 109(2), 151–160. 10.1161/CIRCRESAHA.110.237339 21617129 PMC3150753

[acel13467-bib-0032] Pires, K. M. , Buffolo, M. , Schaaf, C. , David Symons, J. , Cox, J. , Abel, E. D. , Selzman, C. H. , Boudina, S. (2017). Activation of IGF‐1 receptors and Akt signaling by systemic hyperinsulinemia contributes to cardiac hypertrophy but does not regulate cardiac autophagy in obese diabetic mice. Journal of Molecular and Cellular Cardiology, 113, 39–50. 10.1016/j.yjmcc.2017.10.001 28987875 PMC5689477

[acel13467-bib-0033] Ren, J. , Yang, L. , Zhu, L. , Xu, X. , Ceylan, A. F. , Guo, W. , & Zhang, Y. (2017). Akt2 ablation prolongs life span and improves myocardial contractile function with adaptive cardiac remodeling: role of Sirt1‐mediated autophagy regulation. Aging Cell, 16(5), 976–987. 10.1111/acel.12616[doi] 28681509 PMC5595687

[acel13467-bib-0034] Rubinsztein, D. C. , Marino, G. , & Kroemer, G. (2011). Autophagy and aging. Cell, 146(5), 682–695. S0092‐8674(11)00828‐2 [pii]; 10.1016/j.cell.2011.07.030[doi] 21884931

[acel13467-bib-0035] Sheng, Y. , Lv, S. , Huang, M. , Lv, Y. , Yu, J. , Liu, J. , & Ding, G. (2017). Opposing effects on cardiac function by calorie restriction in different‐aged mice. Aging Cell, 16(5), 1155–1167. 10.1111/acel.12652[doi] 28799249 PMC5595678

[acel13467-bib-0036] Symons, J. D. , Hu, P. , Yang, Y. , Wang, X. , Zhang, Q. J. , Wende, A. R. , Sloan, C. L. , Sena, S. , Abel, E. D. , Litwin, S. E. (2011). Knockout of insulin receptors in cardiomyocytes attenuates coronary arterial dysfunction induced by pressure overload. American Journal of Physiology‐Heart and Circulatory Physiology, 300(1), H374–381. 10.1152/ajpheart.01200.2009 20971769 PMC3023253

[acel13467-bib-0037] Symons, J. D. , Rendig, S. V. , Stebbins, C. L. , & Longhurst, J. C. (2000). Microvascular and myocardial contractile responses to ischemia: influence of exercise training. Journal of Applied Physiology (1985), 88(2), 433–442. 10.1152/jappl.2000.88.2.433 10658008

[acel13467-bib-0038] Taneike, M. , Nishida, K. , Omiya, S. , Zarrinpashneh, E. , Misaka, T. , Kitazume‐Taneike, R. , Austin, R. , Takaoka, M. , Yamaguchi, O. , Gambello, M. J. , Shah, A. M. , Otsu, K. (2016). mTOR hyperactivation by ablation of tuberous sclerosis complex 2 in the mouse heart induces cardiac dysfunction with the increased number of small mitochondria mediated through the down‐regulation of autophagy. PLoS One, 11(3), e0152628. 10.1371/journal.pone.0152628 27023784 PMC4811538

[acel13467-bib-0039] Taneike, M. , Yamaguchi, O. , Nakai, A. , Hikoso, S. , Takeda, T. , Mizote, I. , Oka, T. , Tamai, T. , Oyabu, J. , Murakawa, T. , Nishida, K. , Shimizu, T. , Hori, M. , Komuro, I. , Takuji Shirasawa, T. S. , Mizushima, N. , Otsu, K. (2010). Inhibition of autophagy in the heart induces age‐related cardiomyopathy. Autophagy, 6(5), 600–606. 11947 [pii]; 10.4161/auto.6.5.11947[doi] 20431347

[acel13467-bib-0040] Terman, A. , & Brunk, U. T. (2005). Autophagy in cardiac myocyte homeostasis, aging, and pathology. Cardiovascular Research, 68(3), 355–365. S0008‐6363(05)00417‐7 [pii]; 10.1016/j.cardiores.2005.08.014[doi] 16213475

[acel13467-bib-0041] Wang, S. , Ge, W. , Harns, C. , Meng, X. , Zhang, Y. , & Ren, J. (2018). Ablation of toll‐like receptor 4 attenuates aging‐induced myocardial remodeling and contractile dysfunction through NCoRI‐HDAC1‐mediated regulation of autophagy. Journal of Molecular and Cellular Cardiology, 119, 40–50. 10.1016/j.yjmcc.2018.04.009 29660306

[acel13467-bib-0042] Wu, B. , Yu, L. , Wang, Y. , Wang, H. , Li, C. , Yin, Y. , Yang, J. , Wang, Z. , Zheng, Q. , Ma, H. (2016). Aldehyde dehydrogenase 2 activation in aged heart improves the autophagy by reducing the carbonyl modification on SIRT1. Oncotarget, 7(3), 2175–2188. 6814 [pii]; 10.18632/oncotarget.6814[doi] 26741505 PMC4823027

[acel13467-bib-0043] Zhang, Q.‐J. , Holland, W. L. , Wilson, L. , Tanner, J. M. , Kearns, D. , Cahoon, J. M. , Pettey, D. , Losee, J. , Duncan, B. , Gale, D. , Kowalski, C. A. , Deeter, N. , Nichols, A. , Deesing, M. , Arrant, C. , Ruan, T. , Boehme, C. , McCamey, D. R. , Rou, J. , … Symons, J. D. (2012). Ceramide mediates vascular dysfunction in diet‐induced obesity by PP2A‐mediated dephosphorylation of the eNOS‐Akt complex. Diabetes, 61(7), 1848–1859. 10.2337/db11-1399 22586587 PMC3379648

[acel13467-bib-0044] Zhang, Y. , Wang, C. , Zhou, J. , Sun, A. , Hueckstaedt, L. K. , Ge, J. , & Ren, J. (2017). Complex inhibition of autophagy by mitochondrial aldehyde dehydrogenase shortens lifespan and exacerbates cardiac aging. Biochimica Et Biophysica Acta (BBA) ‐ Molecular Basis of Disease, 1863(8), 1919–1932. 10.1016/j.bbadis.2017.03.016 28347844

