# Supplementary figures and images for: Late‐in‐life treadmill training rejuvenates autophagy, protein aggregate clearance, and function in mouse hearts
Source: Aging Cell. 2021 Sep 23;20(10):e13467. doi: 10.1111/acel.13467 (PMC8520717; doi:10.1111/acel.13467)

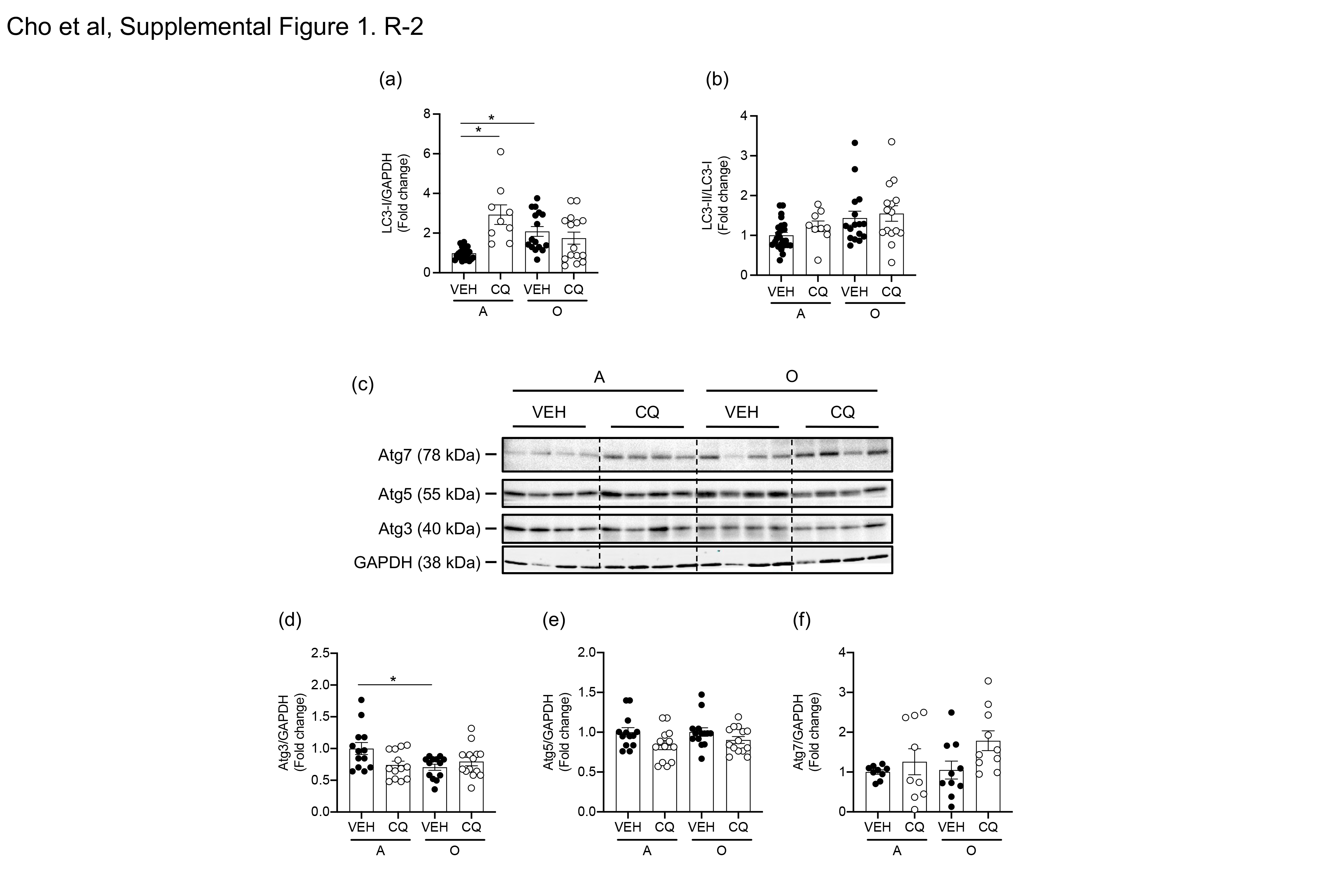

Supplement: Supplementary file 1 — Figure S1 [file ACEL-20-e13467-s001.tif]

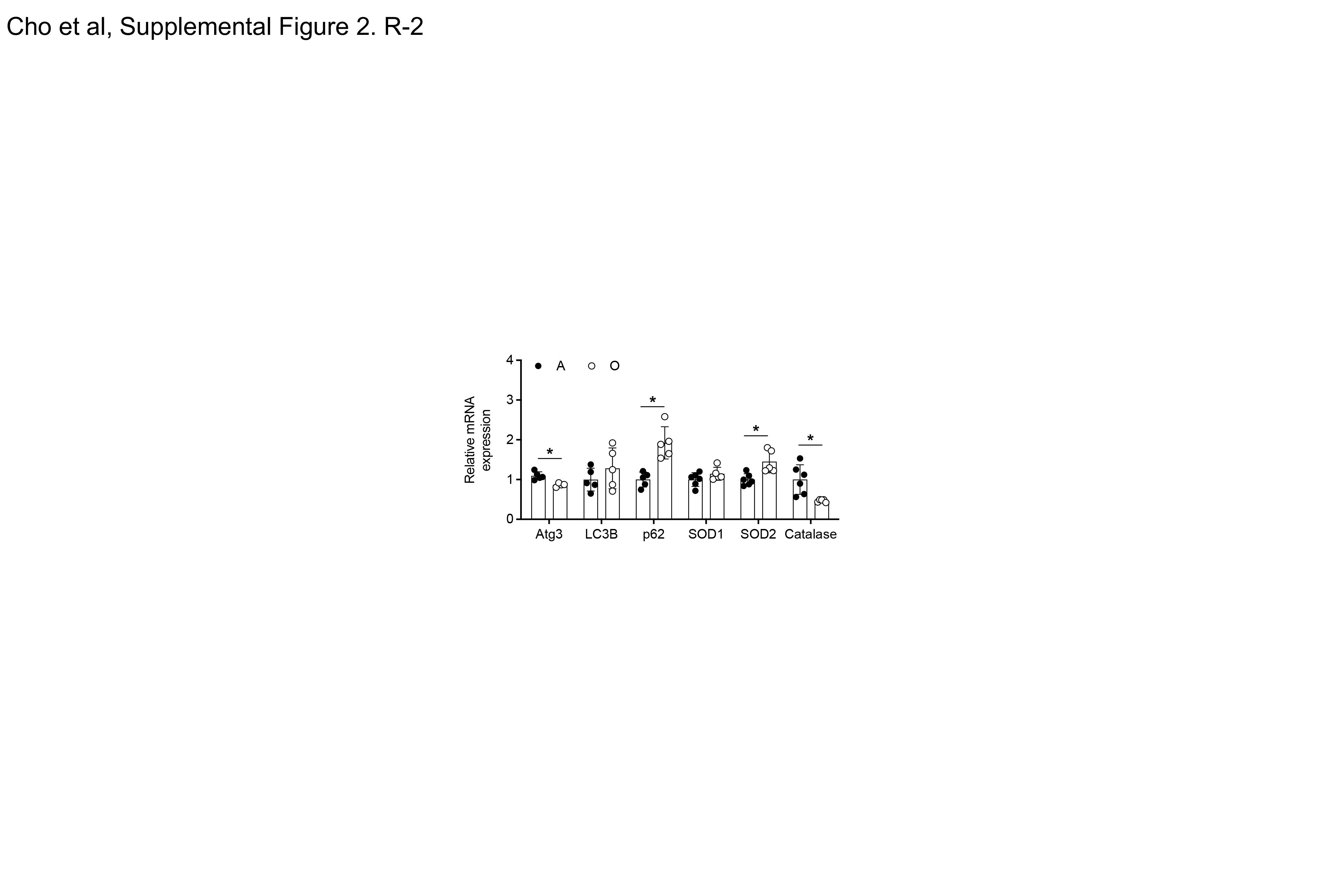

Supplement: Supplementary file 2 — Figure S2 [file ACEL-20-e13467-s011.tif]

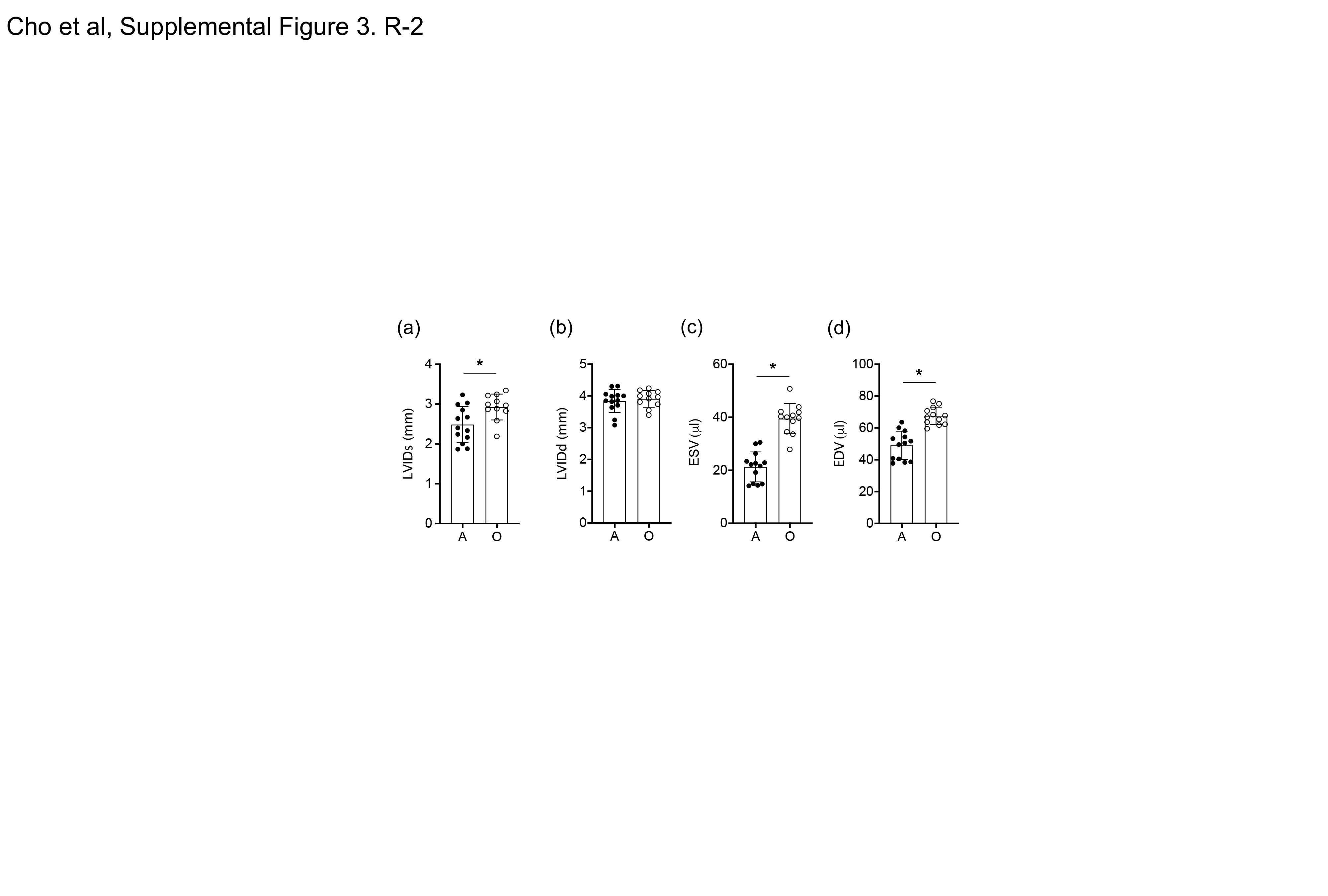

Supplement: Supplementary file 3 — Figure S3 [file ACEL-20-e13467-s002.tif]

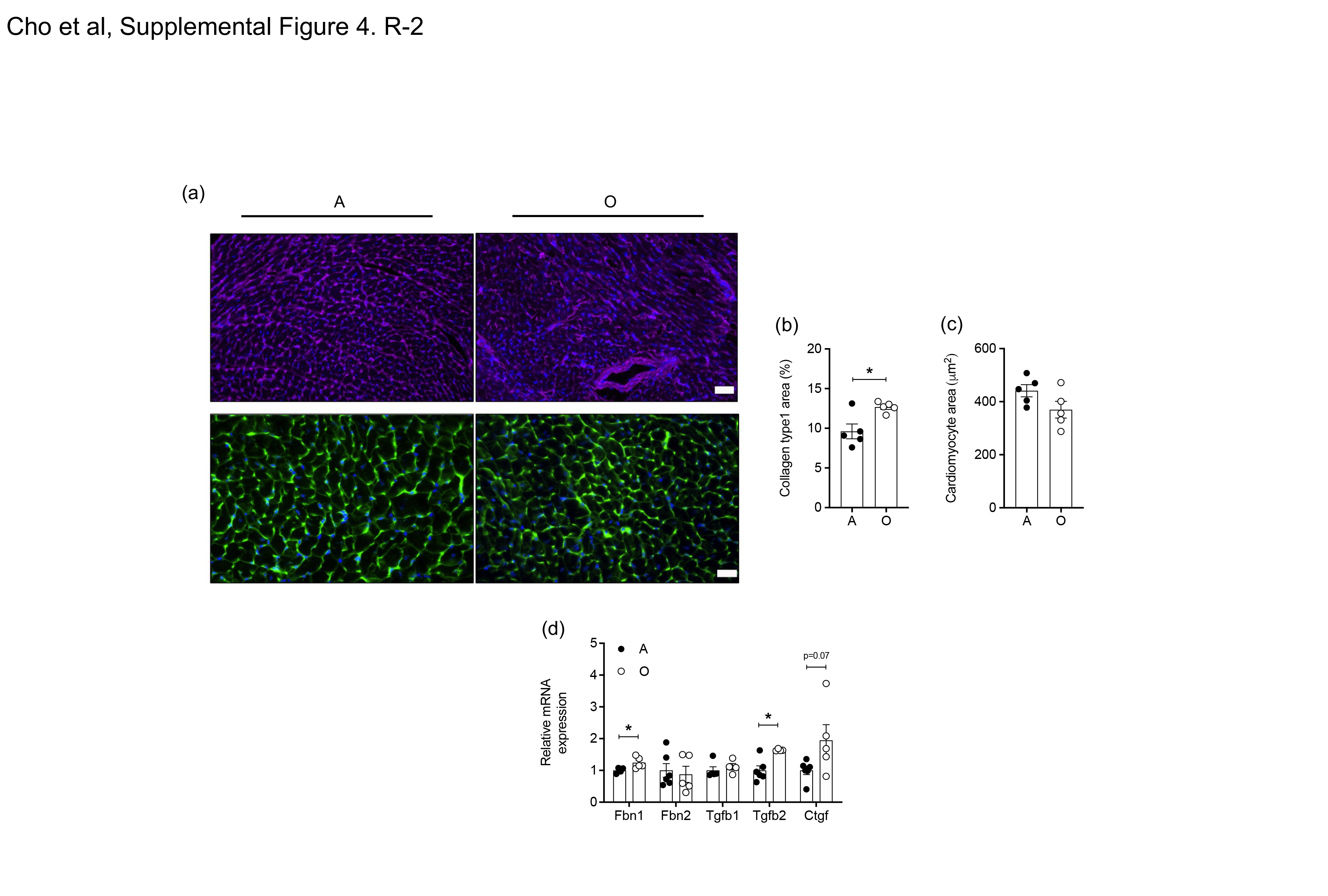

Supplement: Supplementary file 4 — Figure S4 [file ACEL-20-e13467-s007.tif]

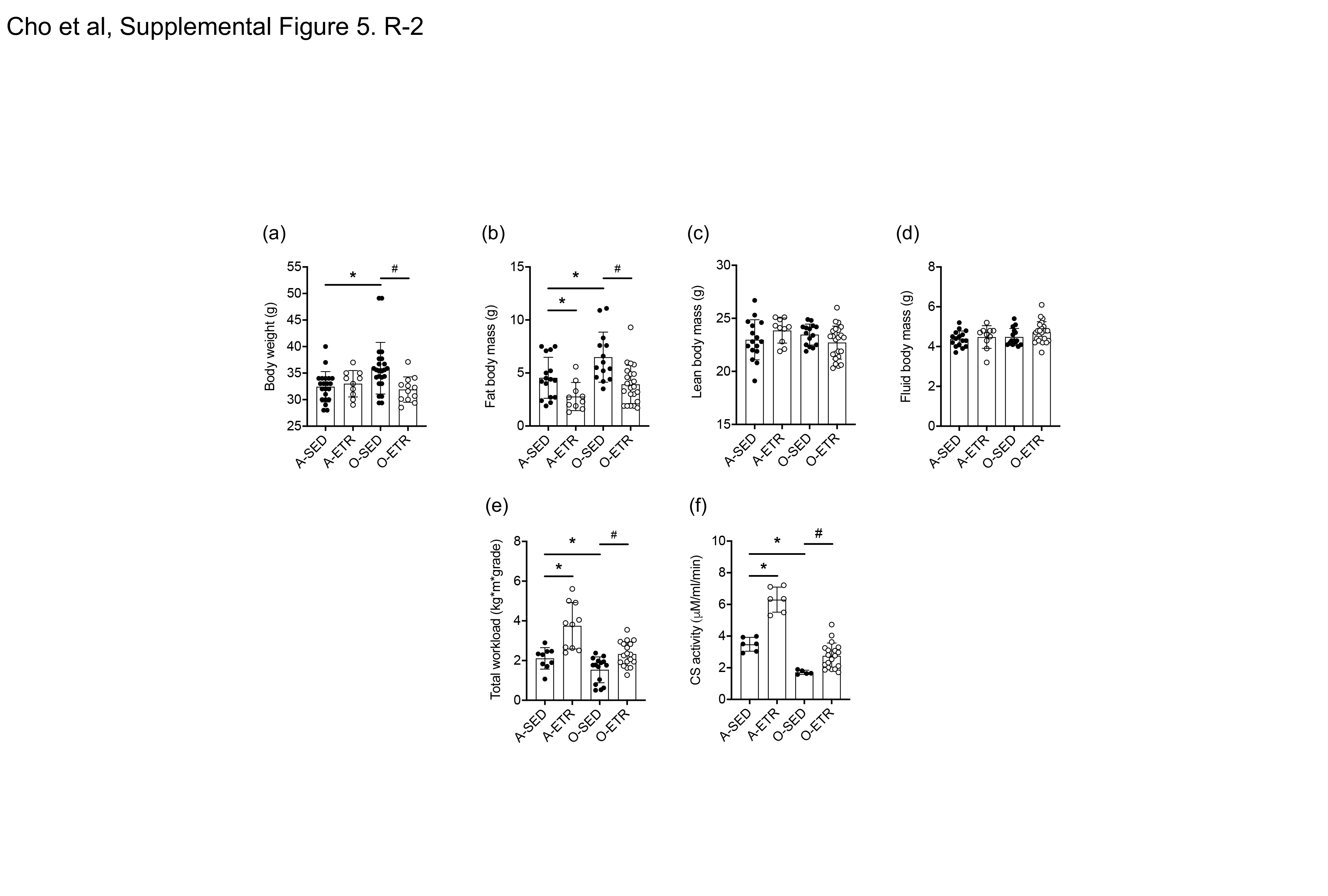

Supplement: Supplementary file 5 — Figure S5 [file ACEL-20-e13467-s016.tif]

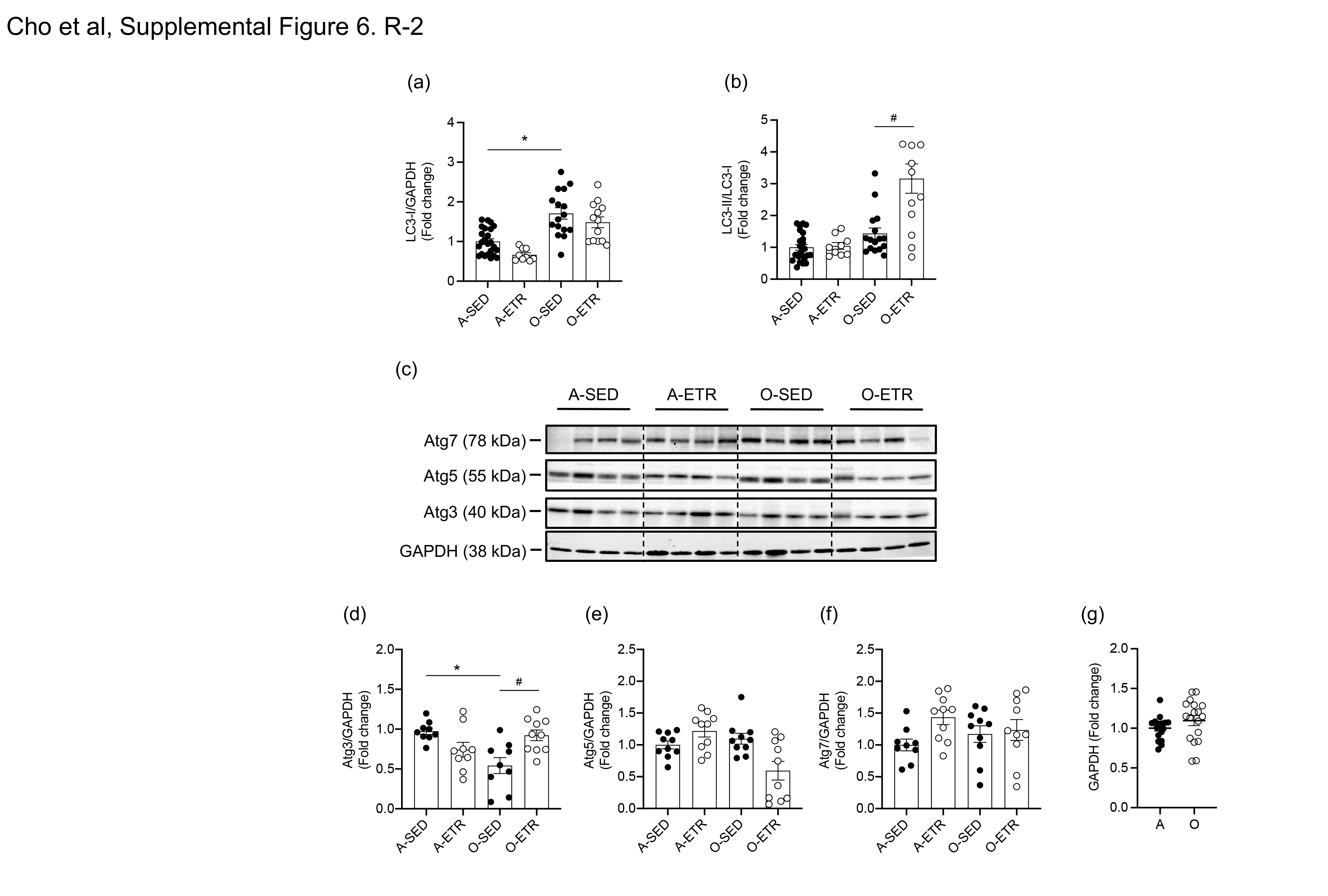

Supplement: Supplementary file 6 — Figure S6 [file ACEL-20-e13467-s005.tif]

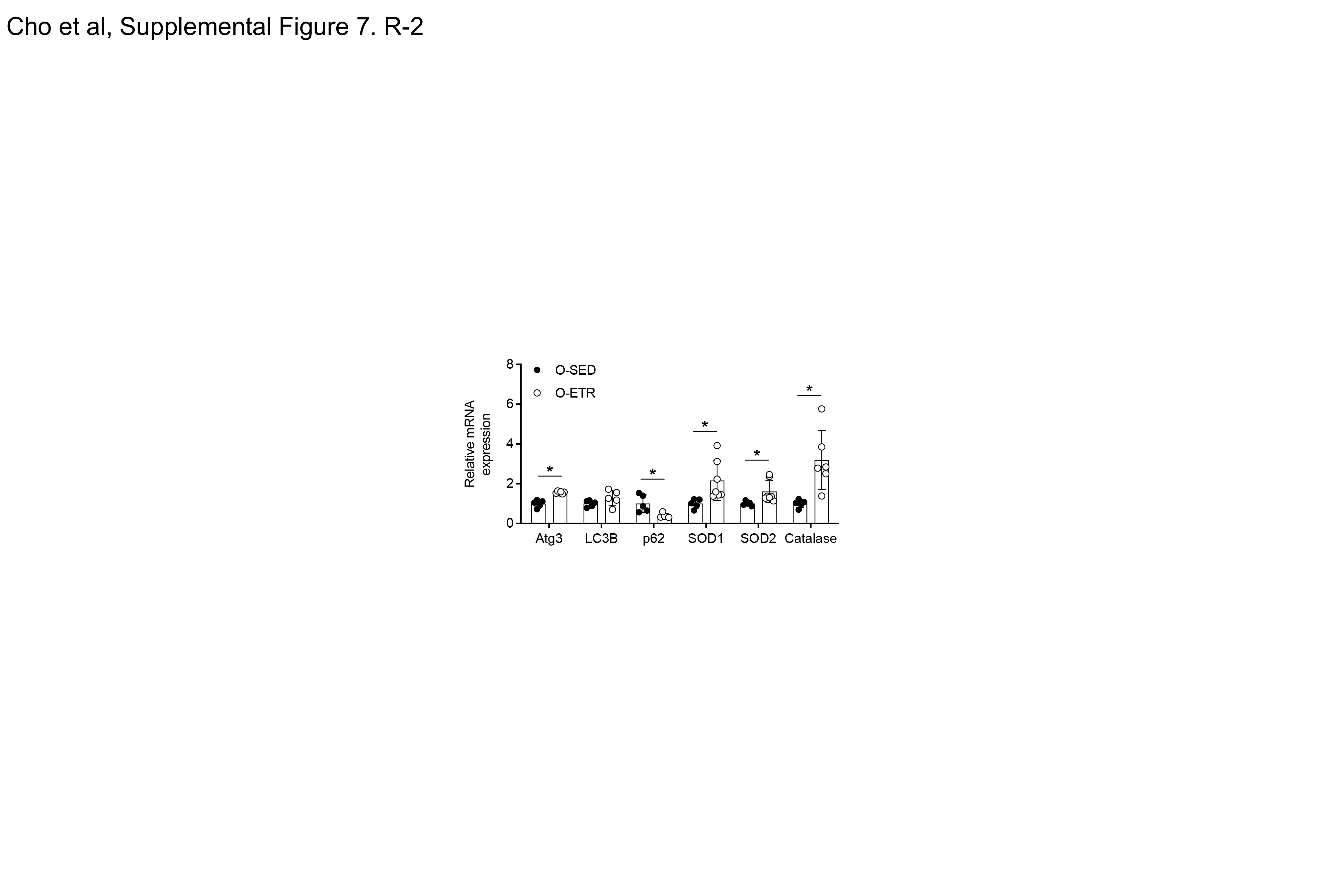

Supplement: Supplementary file 7 — Figure S7 [file ACEL-20-e13467-s014.tif]

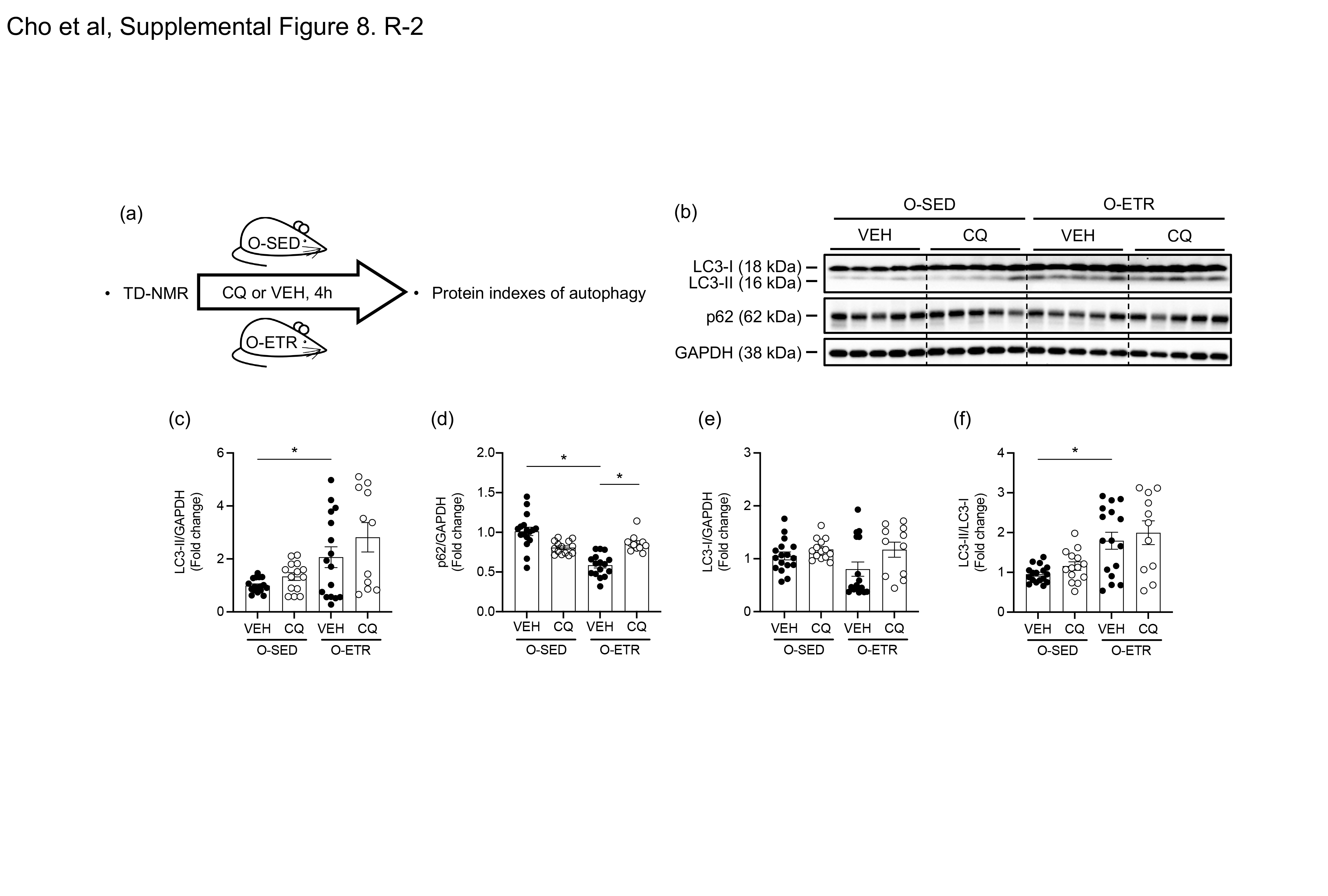

Supplement: Supplementary file 8 — Figure S8 [file ACEL-20-e13467-s015.tif]

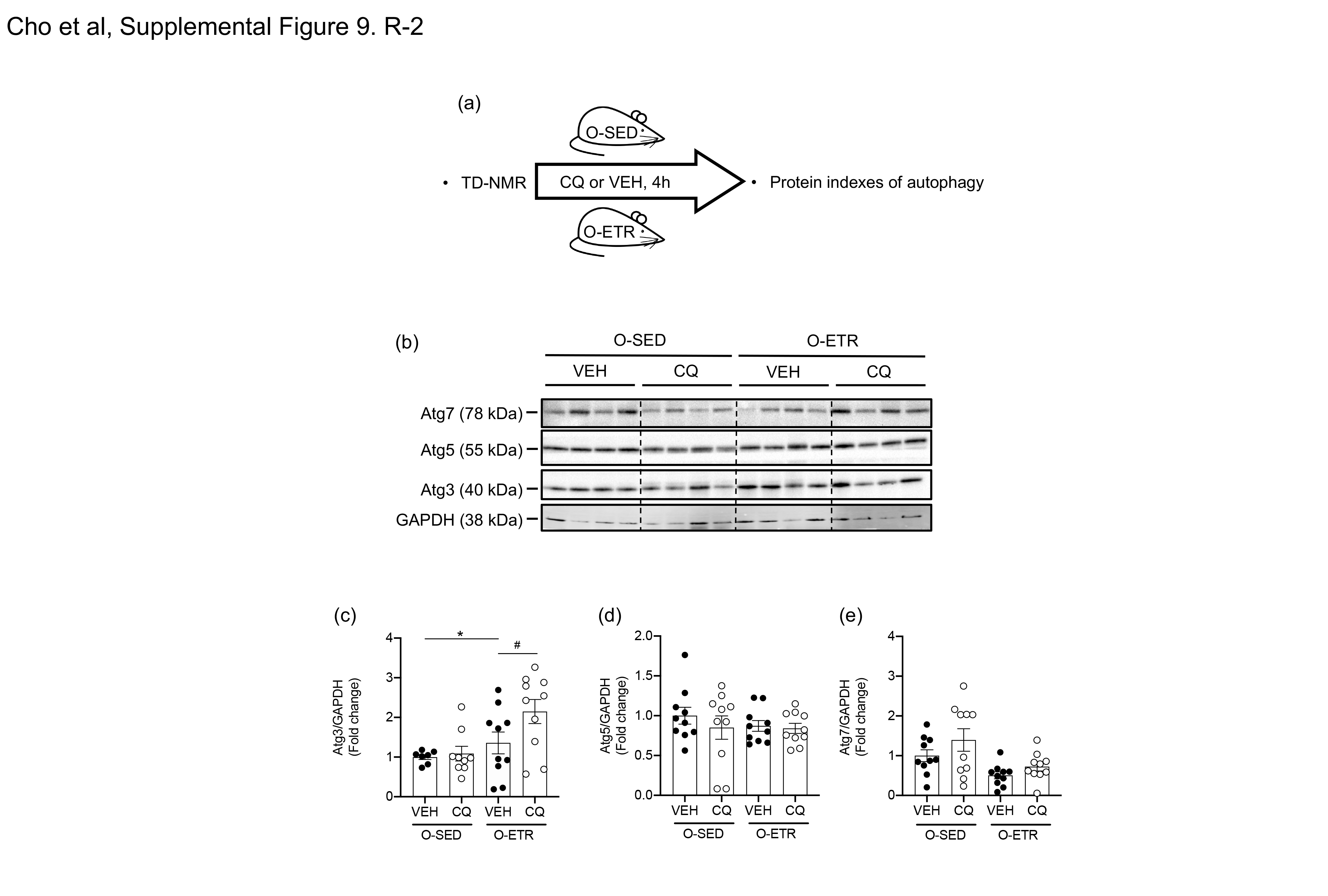

Supplement: Supplementary file 9 — Figure S9 [file ACEL-20-e13467-s013.tif]

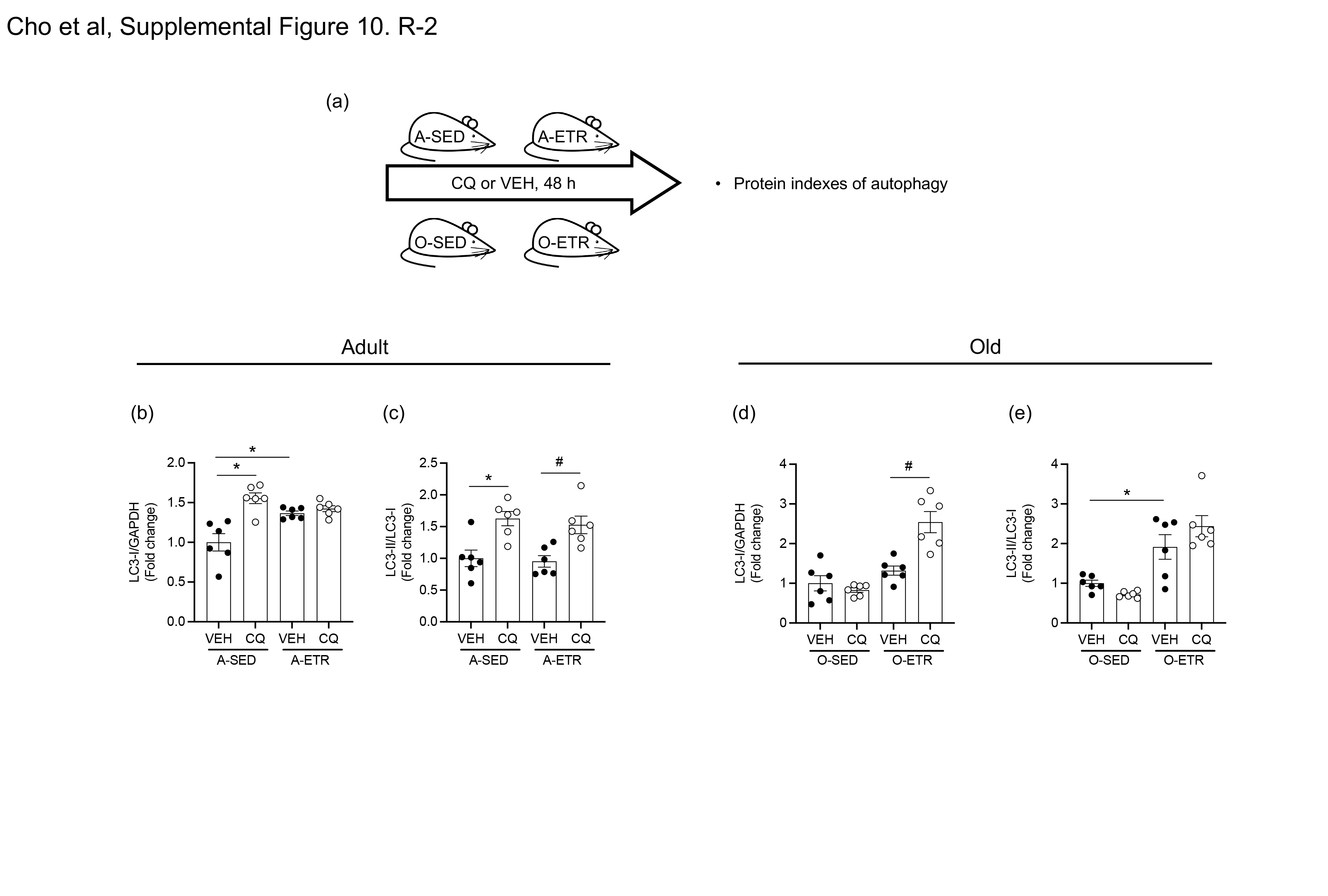

Supplement: Supplementary file 10 — Figure S10 [file ACEL-20-e13467-s008.tif]

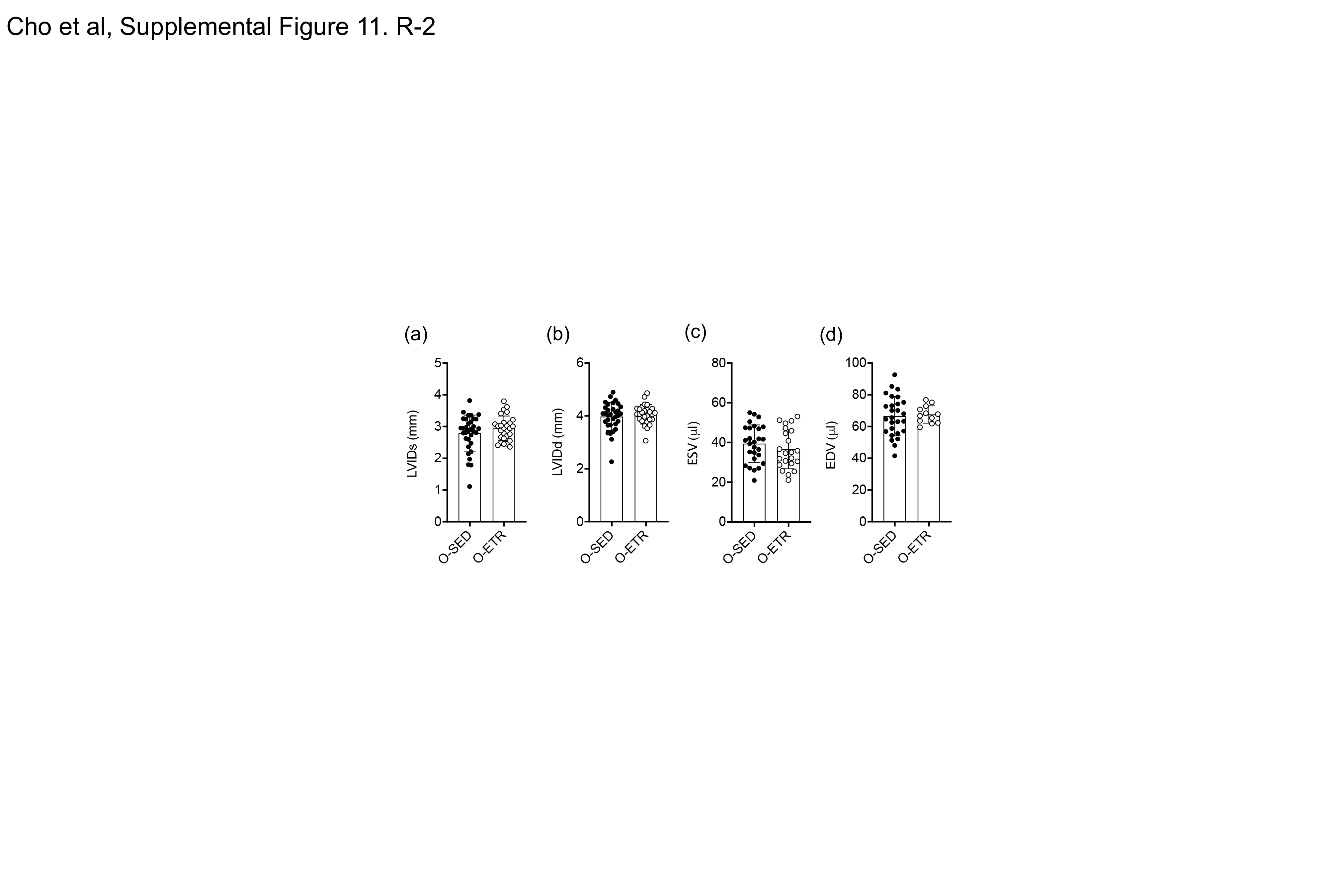

Supplement: Supplementary file 11 — Figure S11 [file ACEL-20-e13467-s006.tif]

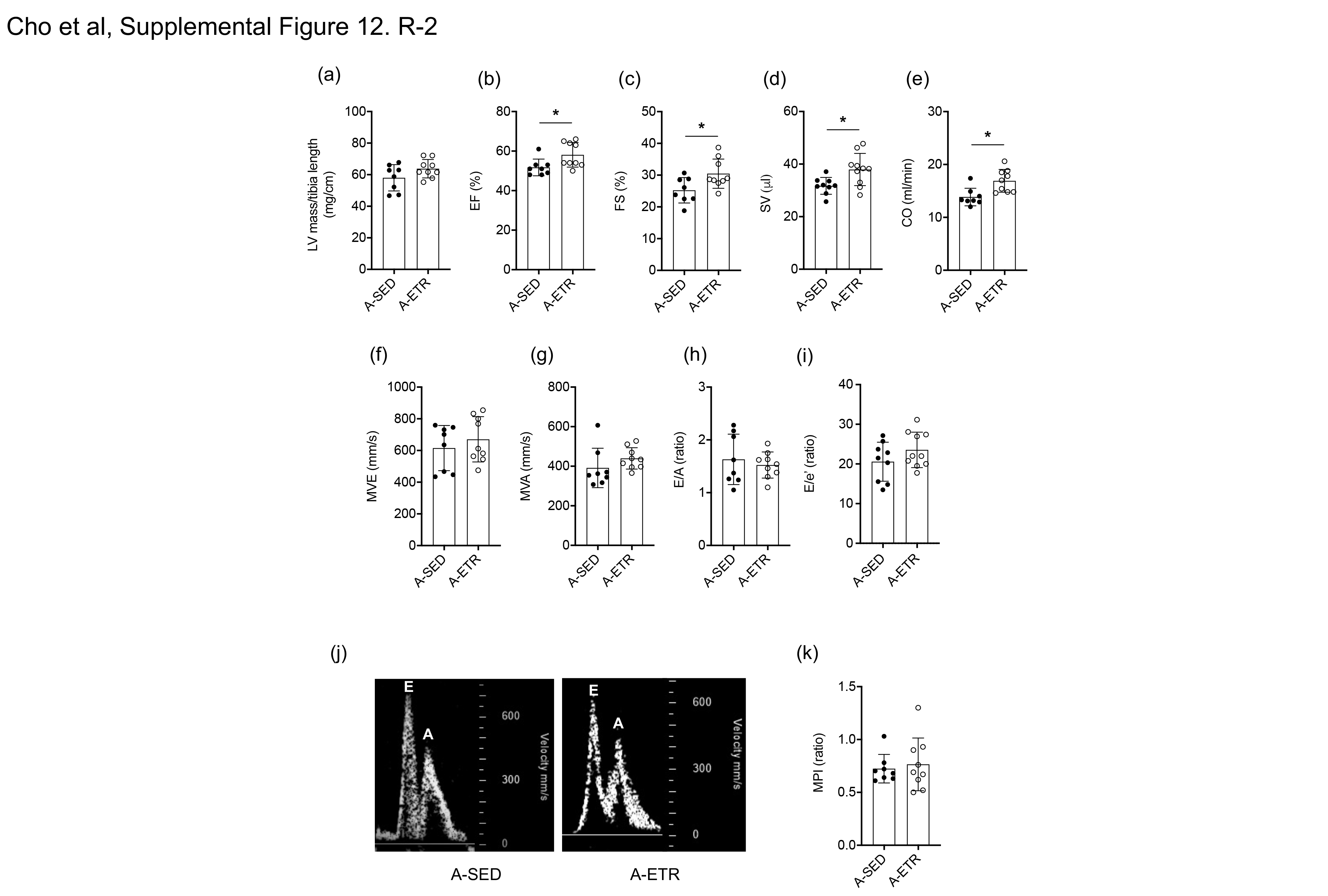

Supplement: Supplementary file 12 — Figure S12 [file ACEL-20-e13467-s004.tif]

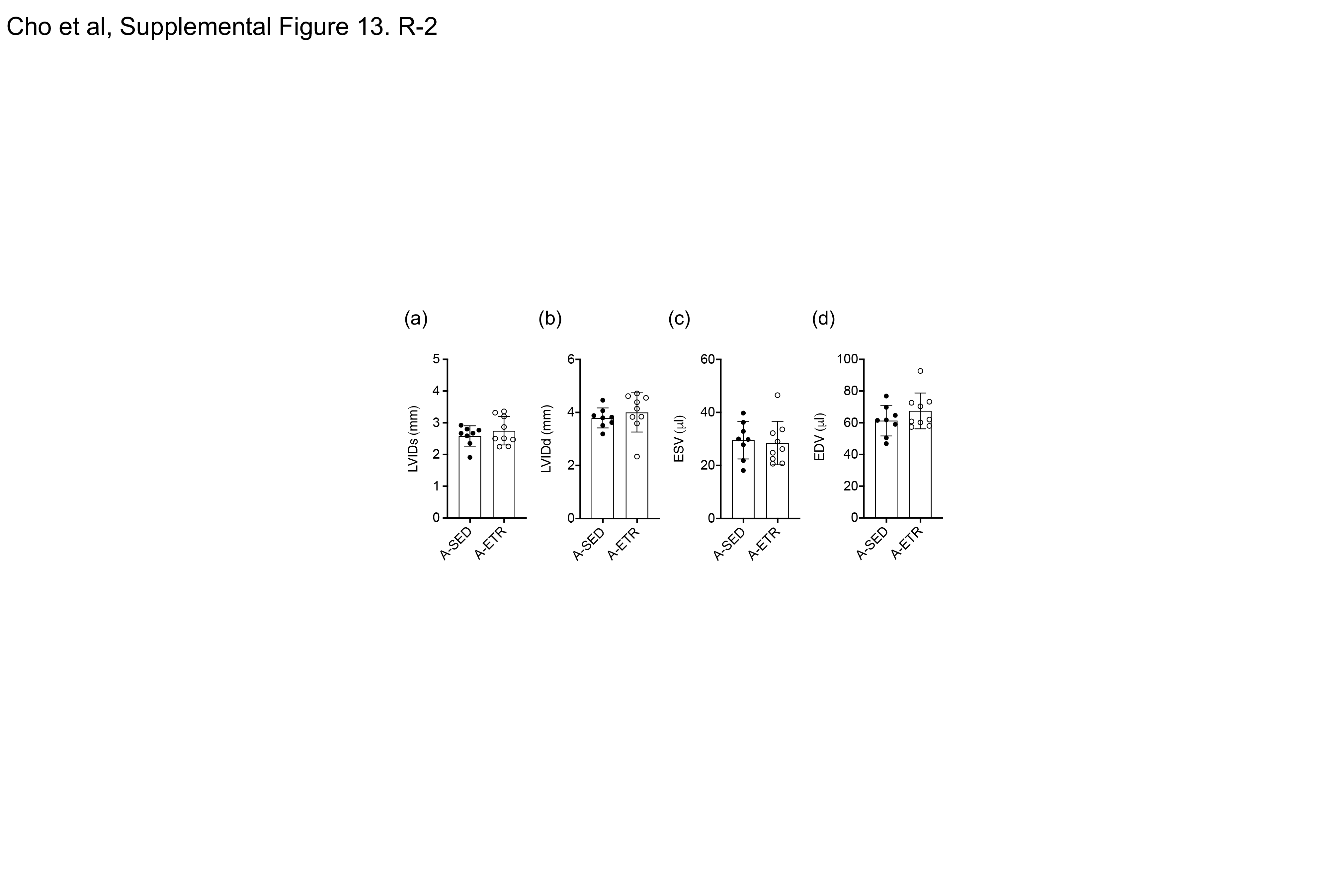

Supplement: Supplementary file 13 — Figure S13 [file ACEL-20-e13467-s003.tif]

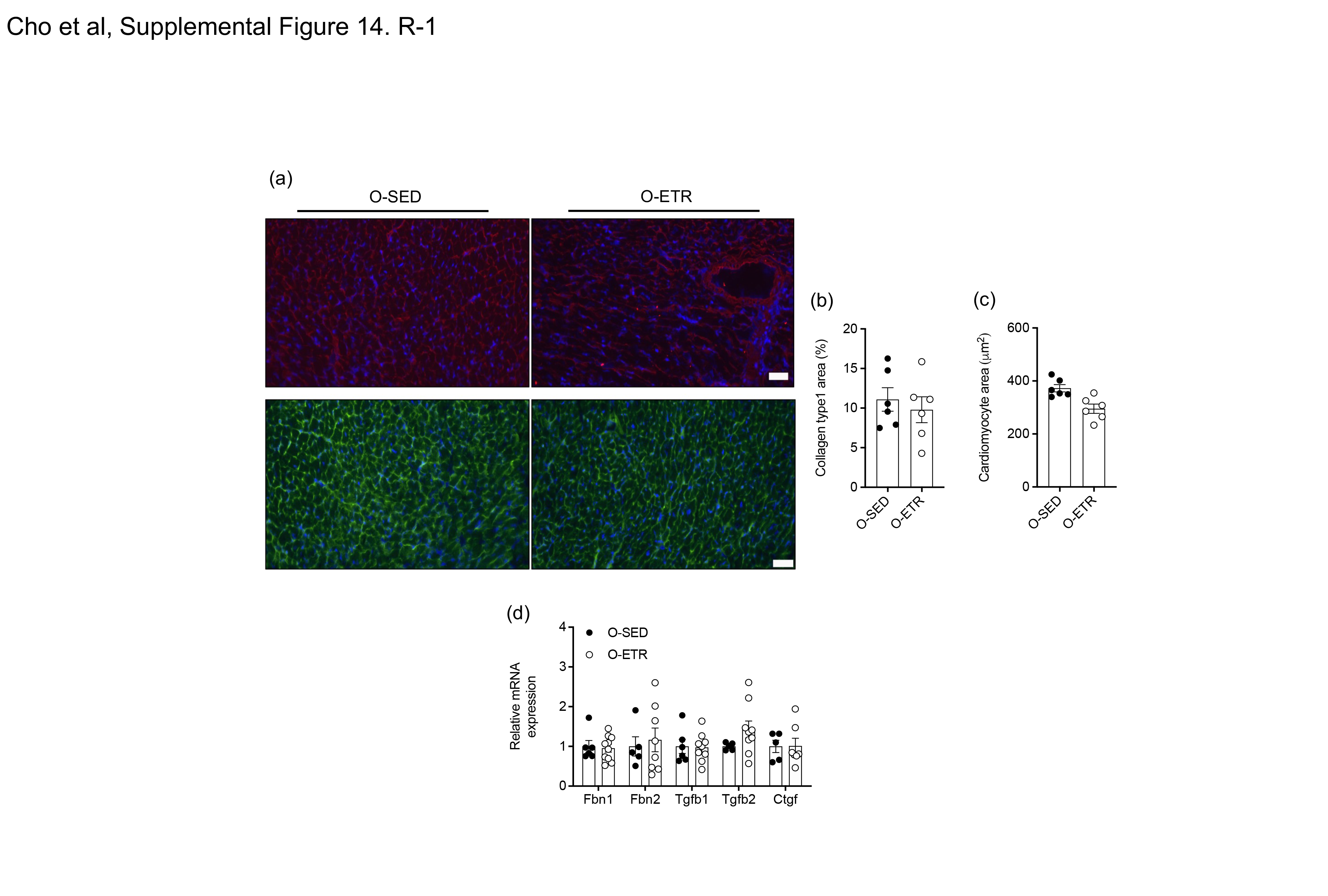

Supplement: Supplementary file 14 — Figure S14 [file ACEL-20-e13467-s012.tif]

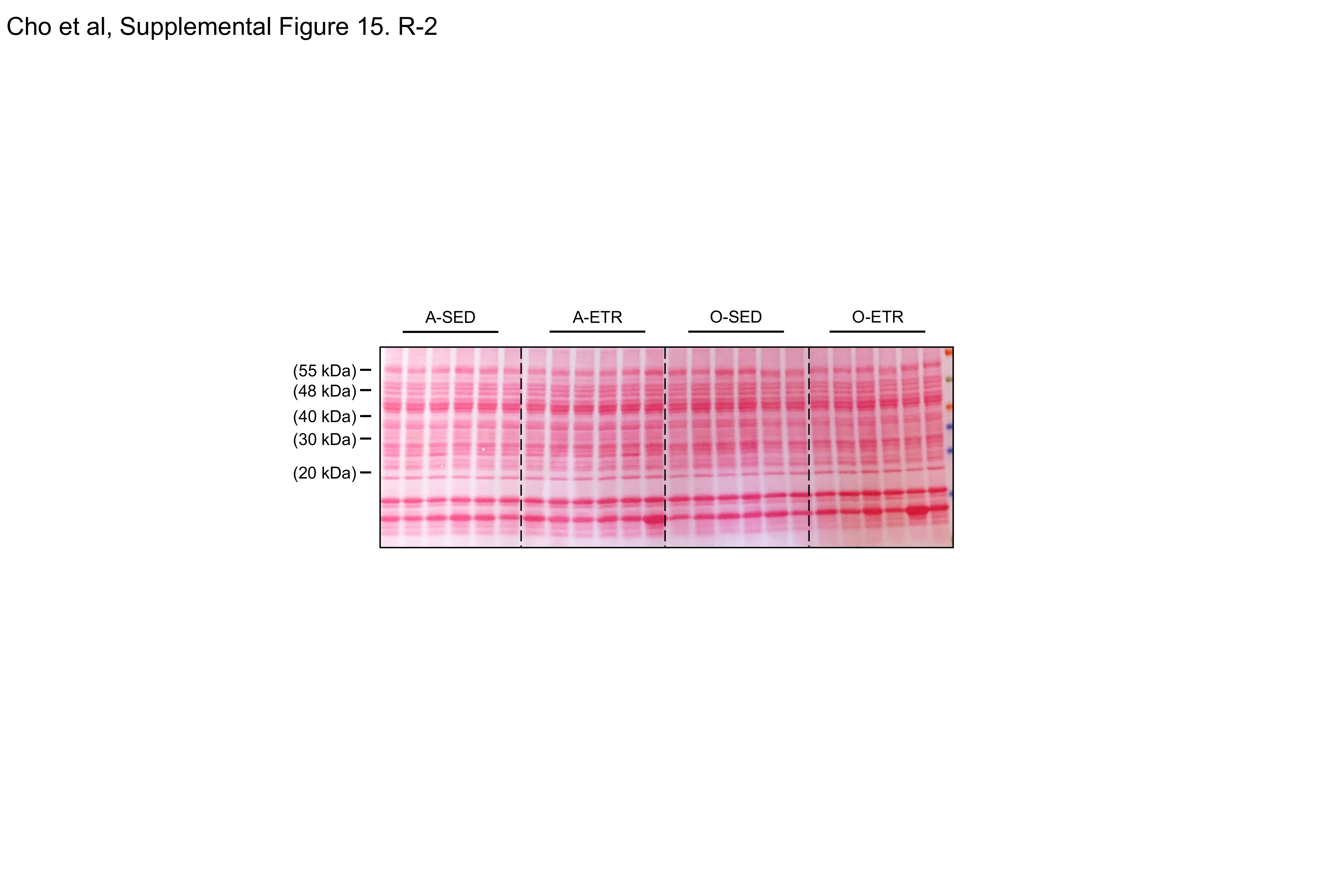

Supplement: Supplementary file 15 — Figure S15 [file ACEL-20-e13467-s009.tif]
