# Supplementary material for: Late‐in‐life treadmill training rejuvenates autophagy, protein aggregate clearance, and function in mouse hearts
Source: Aging Cell. 2021 Sep 23;20(10):e13467. doi: 10.1111/acel.13467 (PMC8520717; doi:10.1111/acel.13467)
Supplement: Supplementary file 16 — Supplementary Material [file ACEL-20-e13467-s010.docx]

**Late-in-life treadmill-training rejuvenates autophagy, protein aggregate clearance, and function in mouse hearts.**

Running head : Training improves cardiac autophagy in old mice

Jae Min Cho^1,2^, Seul-Ki Park^1,2^, Rajeshwary Ghosh^1,2^, Kellsey Ly^1,2^, Caroline Ramous^1,2^, Lauren Thompson^1,2^, Michele Hansen^1,2^, Maria Sara de Lima Coutinho Mattera^6^, Karla Maria Pires^1,2^, Maroua Ferhat^1,2^, Kevin J Whitehead^1,3,4,5^, Kandis Carter^1,3^, Márcio Buffolo^1,2^, Sihem Boudina^1,2,3*^, J David Symons^1,2,3*^

^1^University of Utah, ^2^Nutrition and Integrative Physiology, ^3^Molecular Medicine Program, ^4^Division of Cardiovascular Medicine and Pediatric Cardiology, ^5^George E Wahlen VA Medical Center, ^6^Sao Paulo State University, School of Dentistry

*Co-corresponding authors

[s.boudina@u2m2.utah.edu](mailto:s.boudina@u2m2.utah.edu)

[j.david.symons@hsc.utah.edu](mailto:j.david.symons@hsc.utah.edu)

**SUPPORTING INFORMATION**

**EXPERIMENTAL PROCEDURES**

*Animals and housing.* Male C57BL/6J mice were obtained from the Jackson Laboratories at 4 months of age and from the National Institute on Aging rodent colony at 18 months of age. Until their use, mice were housed four per cage at the AALAC-approved Comparative Medicine Center at the University of Utah under specific guidelines which included a 12-h light and 12-h dark-cycle sand temperature-controlled environment (22-23°C). Mice were given standard rodent chow and water ad libitum and animals were checked daily by vivarium staff. Under the guidance and regulation of the Institutional Animal Care and Use Committee at the University of Utah, all mice were handled according to approved procedures documented in protocol number 19-07010.

*Cardiac autophagy.* We hypothesized that static (i.e., basal) cardiac autophagy and / or autophagosome formation (i.e., autophagic flux) are repressed in hearts from 24-month vs. 8-month old mice. A schematic is shown in Figure 1a. Lean mass, fat mass, and fluid mass were assessed in all mice using time-domain-nuclear magnetic resonance (TD-NMR; Bruker minispec, Bruker Biospin Corporation)(Bharath et al., 2017; Bharath et al., 2015). Twenty-four h later, the lysosomal acidification inhibitor chloroquine (CQ; 75 mg IP/g lean body mass) or vehicle-control (phosphate-buffered saline; PBS)(Gottlieb, Andres, Sin, & Taylor, 2015; Pires et al., 2017) was administered to separate cohorts of older and adult mice. Four h later, mice were anesthetized using 2-5% inhaled isoflurane combined with 100% oxygen. When a stable plane of anesthesia was attained, the chest was opened using aseptic procedures, the heart was exposed, excised, and used to assess protein indexes of autophagy (Bharath et al., 2017), ubiquitinated proteins, and 4-hydroxy-2-nonenal (4-HNE), an **α, β-unsaturated hydroxyalkenal that is an estimate of lipid peroxidation.** Protein isolation and immunoblotting analyses were performed as described by us (Bharath et al., 2017; Bharath et al., 2015; Symons et al., 2011; Zhang et al., 2012). In brief, total protein from each heart was separated by SDS-PAGE (4-20%), transferred onto polyvinylidene difluoride (ThermoFisher), and probed with LC3-II, LC3-I, p62/SQSTM1, total-ubiquitin, 4-HNE, and GAPDH primary antibodies. Alexa Fluor anti-rabbit 680 (Invitrogen) and anti-mouse 800 (VWR International) served as secondary antibodies. Fluorescence was quantified using the Odyssey imager (LI-COR Biosciences). In additional cohorts of mice a longer duration of CQ was performed i.e., 48 h (30 mg/kg), 24 h (30 mg/kg) and 4 h (50 mg/kg) prior to tissue collection (Figure 4a)(Ju, Varadhachary, Miller, & Weihl, 2010).

*Cardiac function.* It was necessary for us to substantiate previous reports that cardiac function observed in adult mice is compromised in older animals (Dai & Rabinovitch, 2009; Dai et al., 2009; Flynn et al., 2013). A schematic is shown in Figure 2a. Separate cohorts of adult and older mice were anesthetized lightly with 1-3% isoflurane anesthesia combined with 100% oxygen while cardiac function was assessed using transthoracic echocardiography (Symons et al., 2011). Acquisitions were made using a Vevo 2100 high-resolution unit equipped with a 22-55 MHz transducer (Visual Sonic)(Pires et al., 2017; Symons et al., 2011). An investigator blinded to mouse age performed the analyses and reduced the data using the Vevo-Strain software / Vevo 2100 imaging system. Parasternal long axis images acquired in B-mode and parasternal short axis images at the level of the papillary muscles acquired in M-mode were captured using an MS 550D transducer. Indices of systolic function [stroke volume (SV), ejection fraction (EF), fractional shortening (FS), cardiac output (CO)], together with end-diastolic volume, end-systolic volume, and end-systolic left ventricular (LV) mass were assessed. Parasternal short-axis measures in M-mode were used to estimate LV anterior wall thickness in diastole and systole, LV internal diameter in diastole and systole, and end-diastolic and end-systolic volumes. Using pulse wave doppler, diastolic function was estimated by measuring passive (i.e., early due to pressure gradient; E) and active (i.e, later due to atrial contraction; A) velocity of blood flow through the mitral valve (MV) and early diastolic annular velocity (e’). These results were used to calculate the E/A and E/e’ ratios. The myocardial performance index (MPI), an indicator of global left ventricular function, was calculated after measuring isovolumetric contraction time (ICT), isovolumetric relaxation time (IRT), and ejection time (ET), as (ICT + IRT) / ET (Goroshi & Chand, 2016). VevoLab analysis software 3.1.0 was used to quantify all measures.

*Histology, morphology, mRNA gene expression.* Twenty-four – 48 h after assessing cardiac function, mice were anesthetized as described, and excised hearts were used to assess protein indexes of autophagy (described earlier), histology, morphology, and mRNA expression of pro-fibrotic and antioxidant genes. Frozen tissue was cut and embedded in optimal cutting temperature (OCT) compound (Fisher Scientific). Five sections (5 µm thick) per sample were placed on glass slides, and stored at -80°C. To assess type 1 collagen, sections were air dried for 60-min, fixed in ice cold acetone (Fisher Scientific) for 10 min, blocked for 60-min in 3% BSA blocking buffer, and stained with anti-Collagen I antibody (1:200, Abcam) at 4°C overnight. Slides were washed three times with 1X PBS and incubated with Alexa Fluor Plus 647 goat anti-Rabbit IgG secondary antibody (1:500, Invitrogen) for 60-min at room temperature. Negative control sections were treated with secondary antibody only. Next, sections were mounted with ProLong™ Gold Antifade Mountant with DAPI (Invitrogen). Ten 20X images were randomly acquired per section with an XM10 Olympus fluorescence camera (Dai & Rabinovitch, 2009). Image quantification was performed using the CellSens Dimension software (Olympus). Percentage of the collagen area in total heart tissue was quantified by ImageJ software (NIH). To assess cardiac myocyte area, sections were stained with wheat germ agglutinin (WGA, Alexa 488, Thermo Fisher) for 60-min at room temperature, followed by DAPI (Alexa 405) for 5-min at room temperature. Ten 20X images were randomly acquired per section with an XM10 Olympus fluorescence camera, and cardiomyocyte cross sectional area (µm^2^) was quantified using CellSens Dimension software (Olympus). To assess mRNA expression of fibrosis, total RNA was extracted from segments of heart from adult and old mice using the RNeasy Mini Kit (Qiagen). (Bharath et al., 2017; Bharath et al., 2015; Symons et al., 2011; Zhang et al., 2012). Total RNA was reverse transcribed to cDNA using the QuantiTect Reverse Transcription Kit (Qiagen). The quantitative gene expression assay was performed with specific primers for fibrillin (Fbn) 1, Fbn2, transforming growth factor-β (Tgfb)1, Tgfb2, connective tissue growth factor (Ctgf), superoxide dismutase (SOD) 1 and SOD2, and catalase, microtubule-associated protein 1A/1B light chain 3B (LC3B), sequestosome 1 (p62), PTEN-induced kinase 1 (Pink1), and Parkin RBR E3 ubiquitin protein ligase (Park2). Relative gene expression was normalized to 18S. Primer sequences are shown in Table S2.

*Exercise training.* Our main focus was to determine whether late-in-life exercise-training improves basal cardiac autophagy and trafficking of the autophagosome to the lysosome to an extent that rejuvenates protein aggregate clearance and recovers cardiac function. A schematic is shown in Figure 3a. Body composition was assessed using TD-NMR in 5-month and 21-month old mice. Twenty-four to 48 h later, mice were familiarized with walking/running on a motorized treadmill (Columbus Instruments) for 3 consecutive days x 5-10 min/day. On day 4, a workload capacity evaluation test was completed on each mouse i.e., 1 min x 5 m/min x 25% grade, followed by 1 m/min increases in speed each min until maximal exercise capacity was achieved. Electrical shocks were not used. Mice were encouraged to run by tapping their rear using test tube cleaning brushes. Total workload was calculated as [body weight (kg) x total running time (min) x final running speed (m/min) x treadmill grade (25%)] (Symons et al., 2011; Symons, Rendig, Stebbins, & Longhurst, 2000).

After all mice finished the workload capacity evaluation, they were separated randomly into groups that did not (adult-SED and old-SED) or did (adult-ETR and old-ETR) complete a 3 mo progressive resistance treadmill-running program. Adult-SED and old-SED mice ran on the treadmill 1 day/week x 5 m/min x 10% grade for 5-min to maintain familiarization. This was required so that SED mice would be able to complete a second workload capacity evaluation test after 3 mo. Adult-ETR and old-ETR mice trained 6 days/week x 3 mo. The initial intensity corresponded to 70% of their workload capacity e.g., 30 min x 11.4 m/min x 5% grade. Over the next 3 mo, running duration, treadmill speed, and/or treadmill grade were increased every four days to 60 min x 17.4 m/min x 15% grade. After 3 mo, body composition, exercise tolerance (maximal workload capacity), and cardiac function were assessed. Each evaluation was separated by 24 h. Twenty-four h after measuring cardiac function, all mice were anesthetized as described, and the heart was obtained to assess protein indexes of autophagy, histology, morphology, mRNA expression of pro-fibrotic (described earlier) and mitophagy-related genes, protein aggregate accumulation, and Complex I-V of the electron transport chain. Soleus muscle was dissected free from both hindlimbs to assess CS enzyme activity (Sigma-Aldrich)(Symons et al., 2000).

*Cardiac protein aggregation.* After determining cardiac protein concentrations (Pierce BCA Protein Assay; ThermoFisher) protein aggregate accrual in the heart was measured using a commercially available kit (Proteostat; Enzo Life Sciences)(Laor et al., 2019). In brief, samples (3 mg/ml) were added to a 96-well plate together with 2 μl of detection buffer and incubated for 5-min in the dark. Fluorescence output was measured at an excitation setting of 550 nm and emission filter of 600 nm (Varioskan Lux, ThermoFisher). Protein aggregation (%) was calculated based on an 8 point standard curve ranging from 0-12% aggregated IgG. Electron microscopy was used as a second approach to estimate cardiac protein aggregates (DiMemmo et al., 2017). Cardiac sections placed in 2.5 % glutaraldehyde at the time of collection were embedded in plastic, sectioned at 0.5 um with glass knives, and further trimmed with a diamond knife. Sections were placed onto 200 mesh copper grids, and subsequently stained with saturated uranyl acetate, which extends preservation, improves contrast of the extracellular matrix, membranes, cytoplasm, and DNA, and facilitates the quantification of protein aggregation (Erickson, Anderson, & Fisher, 1987). Three fields of view were chosen based on the quality of the images. Images were photographed at 1100x, 2700x, 4400x, and 11000x magnification (FEI Tecnai T-12, ThermoFisher). Results were calculated by quantifying the number of protein aggregate clusters and mitochondria at 2700x magnification due to the clear and detailed images obtained using NIH Image J software.

*Complex I-V protein expression.*  Protein expression of Complex I-V of the electron transport chain was assessed in adult and older mice that did or did not train. Protein isolation and immunoblotting analyses were performed as described earlier. Briefly, proteins were separated by SDS-PAGE (4-20%), transferred onto polyvinylidene difluoride (ThermoFisher), stained with Ponceau S to confirm equal loading, and probed with probed with an antibody cocktail for NDUFB8 (Complex 1), SDHB (Complex II), UQCRC2 (Complex III), MTCO1 (Complex IV), and ATP5A (Complex V).

**Supplementary Figure legends**

**Supplementary Figure 1.** ***Atg3 protein expression is lower in myocardium from older vs. adult mice.*** These data supplement main Figure 1. A and O mice were treated with CQ (75 mg / kg lean muscle mass) or VEH and hearts were obtained 4 h later. Representative images (Main figure 1b) and mean data ± standard error (a, b) are shown. Aging increased LC3-I (a) but not LC3-II:LC3-I (b). CQ increased LC3-I in hearts from A but not O mice (a). CQ did not influence LC3-II:LC3-I in either group. Representative images (c) and mean data ± standard error are shown for protein expression of Atg3 (d), Atg5 (e), and Atg7 (f). Atg3 protein expression was lower in hearts from O vs. A mice, whereas Atg7 and Atg5 were similar between groups. Atg3, Atg5, and Atg7 were refractory to CQ treatment in A and O mice. For (a, b), n=9-24, *p<0.05 vs A-VEH. For (c-f), n=9-13, *p<0.05 vs A-VEH. Data are expressed as fold change relative to values obtained from A mice.

**Supplementary Figure 2. *mRNA expression in hearts from adult and older mice.*** These data supplement main Figure 1. Cardiac autophagy (Atg3, LC3B, and p62) and antioxidant (SOD1, SOD2, and catalase) mRNA expression was assessed and normalized to 18S. Atg3 and catalase were lower whereas p62 and SOD2 were higher in hearts from O vs A mice. LC3B and SOD1 were similar between groups. n=4-6, *p<0.05 vs A. Data are expressed as fold change relative to values obtained from A mice.

**Supplementary Figure 3. *Cardiac function is impaired in older vs. adult mice.*** These data supplement main Figure 2. Transthoracic echocardiography was performed on A and O mice. Mean data ± standard error are shown (a-d). (a) Left ventricular (LV) internal dimension in systole (LVIDs, mm), (c) end-systolic volume (ESV, µl), and (d) end- diastolic volume (EDV, µl) were higher in O vs. A mice, whereas (b) LV internal dimension in diastole (LVIDd, mm) was not different between groups. For (a-d) n=12-13, *p<0.05 vs A.

**Supplementary Figure 4. *Fibrosis exists in older vs. adult mouse hearts.*** These data supplement main Figure 2. Twenty-four h after completing transthoracic echocardiography in A and O mice, hearts were excised and prepared using procedures detailed in the text. Representative immunohistochemistry images (a) and mean data ± standard error indicate type 1 collagen (%, b) was greater in hearts from O vs. A mice, whereas cardiomyocyte cross-sectional area (μm^2^) was similar between groups (c). Scale bar = 50 μm (top) and 20 μm (bottom). mRNA expression of fibrosis-related genes assessed via qPCR and normalized to 18S are shown (d). Fibrillin (Fbn) 1, transforming growth factor-β (Tgfb) 2, and connective tissue growth factor (Ctgf) were elevated in hearts from O vs. A mice, whereas Fbn2 and Tgfb1 were similar between groups. For (d) data are expressed as fold change relative to values of A. For (b, c), n=5 mice per group, n = 5 fields of view. For (d), n=4-6 per group. *p<0.05 vs A.

**Supplementary Figure 5. *Exercise-training is efficacious in adult and older mice.*** These data supplement main Figure 3. A and O mice did (ETR) or did not (SED) complete 12-weeks of treadmill-running. Data shown in (a-f) are from A and O mice at 8 mo and 24 mo, respectively. O-SED mice displayed (a) greater body mass and (b) fat mass, together with (e) reduced exercise capacity and (f) soleus muscle CS enzyme activity, vs. results from A-SED mice, whereas lean mass (c) and fluid mass (d) were similar between groups. Exercise training reduced fat mass (b), and increased exercise capacity (e) and CS activity (f) in A and O mice. Data in (a-f) are mean ± standard error. For (a-d), n=10-24; (e), n=9-18; (f), n=5-19. *p<0.05 vs A-SED; #p<0.05 vs O-SED.

**Supplementary Figure 6. *Atg3 protein expression is increased by late-in-life exercise training.*** These data supplement main Figure 3. A and O mice did (ETR) or did not (SED) complete 12-weeks of treadmill-running. At least 24 h following the last exercise bout, hearts were prepared for immunoblotting. Representative images (main Figure 3b) and mean data ± standard error are shown (a, b). LC3-I increased in hearts from O-SED vs. A-SED mice (a). LC3-II : LC3-I increased in O-ETR vs. O-SED mice (b). Representative images (c) and mean data ± standard error (d-f) are shown. Atg3 was lower in hearts from O-SED vs. A-SED mice, but expression was restored by exercise training (d). Atg5 (e) and Atg7 (f) protein expression was similar between groups regardless of exercise training. (g) GAPDH was not different between A and O mice shown in Figures 1 and 3, indicating its stability between 8 and 24 mo (g).These data recapitulate findings from A and O mice shown in Supplementary Figure 1. Results in (a-f) are expressed as fold change relative to values obtained from A-SED mice. For (a, b), n=9-24. For (d-f), n=9-10. For (g), n=18. *p<0.05 vs A-SED; #p<0.05 vs O-SED.

**Supplementary Figure 7. *mRNA expression of Atg3, p62, SOD1, SOD2, and catalase are altered in hearts from O-ETR vs O-SED mice.*** These data are supplemental to main Figure 3. Cardiac autophagy (Atg3, LC3B, and p62) and antioxidant (SOD1, SOD2, and catalase) mRNA expression were assessed and normalized to 18S. mRNA expression of Atg3, SOD1, SOD2 and catalase increased and p62 decreased in O-ETR vs. O-SED mice, whereas LC3B was similar between groups. n=4-6 per group, *p<0.05 vs O-SED. Data are expressed as fold change relative to values obtained from O-SED mice.

**Supplementary Figure 8.** ***Steady-state autophagy and autophagic flux is improved by late-in-life exercise training in mouse hearts.*** These data supplement main Figure 4. (a) O mice that did (ETR) or did not (SED) complete 12-weeks of exercise training were treated with 4 h CQ or VEH and hearts were obtained 4 h later. Representative images (b) and mean data ± standard error are shown (c-f). LC3-I was similar among groups (c). LC3-II (d) and LC3-II:LC3-I (e) was higher, whereas p62 was lower (f), in hearts from O-ETR-VEH vs. O-SED-VEH mice. These findings support that steady state autophagy is improved by late-in-life exercise training. 4 h CQ treatment increased p62 in myocardium from ETR but not SED mice (f), whereas LC3-I, LC3-II, and LC3-II:LC3-I were not affected (c-e). Results concerning p62 support that autophagic flux is improved by late-in-life exercise training. For (c-f), n=11-17 *p<0.05 vs O-SED; #p<0.05 vs O-ETR. Data are expressed as fold change relative to values obtained from O-SED-VEH mice.

**Supplementary Figure 9.** ***Atg3 protein expression is increased by late-in-life exercise training.*** These data supplement main Figure 4. (a) O mice that did (ETR) or did not (SED) complete 12-weeks of exercise training were treated with 4 h CQ or VEH and hearts were obtained 4 h later. Representative images (a) and mean data ± standard error are shown (b-d). Atg3, but not Atg5 or Atg7, increased in O-ETR vs. O-SED mice. 4 h CQ administration increased Atg3 in O-ETR but not O-SED mice. Atg5 and Atg7 were not responsive to 4 h CQ treatment in O-ETR or O-SED mice. For panels (b-d), n=7-10, *p<0.05 vs O-SED; #p<0.05 vs O-ETR. Data are expressed as fold change relative to values obtained from O-SED mice.

**Supplementary Figure 10.** ***Autophagic flux is improved by late-in-life exercise training in mouse hearts.*** These data supplement main Figure 4. (a) A and O mice that did (ETR) or did not (SED) complete 12-weeks of exercise training. Mice were treated treated with VEH or CQ 48 h, 24 h, and 4 h prior to tissue collection. Representative images for A and O mice are shown in main Figure 4b, c, respectively, and mean data ± standard error are shown (b-e). CQ increased LC3-I and LC3-II:LC3-I in hearts from A-SED (b, c) but not O-SED (d, e) mice. CQ increased LC3-II:LC3-I (c) but not LC3-I (b) in hearts from A-ETR mice. CQ did not influence LC3-II:LC3-I in hearts from O-ETR mice (e) but LC3-I was elevated in a robust manner (d). Together with data shown in main Figure 4, autophagic flux is improved by late-in-life exercise training in mouse hearts. For (b, c) n=6. *p<0.05 vs A-SED-VEH; #p<0.05 vs A-ETR-VEH. Data are expressed as fold change relative to values obtained from A-SED-VEH mice. For (c, d), n=6. *p<0.05 vs O-SED-VEH; #p<0.05 vs O-ETR-VEH. Data are expressed as fold change relative to values obtained from O-SED-VEH mice.

**Supplementary Figure 11. *Cardiac function in trained and untrained older mice.*** These data supplement main Figure 5. O mice did (ETR) or did not (SED) complete 12-weeks of treadmill-running. At least 24 h following the last exercise bout, transthoracic echocardiography was completed. Mean data ± standard error are shown (a-d). (a) Left ventricular (LV) internal dimension in systole (LVIDs, mm) and (b) diastole (LVIDd, mm), and (c) end-systolic (ESV, µl) and (d) end-diastolic (EDV, µl) volume were similar in O-SED and O-ETR mice. For (a-d), n=27-33, *p<0.05 vs O-SED.

**Supplementary Figure 12.** ***Exercise-training improves systolic myocardial function in adult mice.*** These data supplement main Figure 5. A mice did (ETR) or did not (SED) complete 12-weeks of treadmill-running. At least 24 h following the last exercise bout transthoracic echocardiography was completed. Mean data ± standard error are shown (a-i and k). Left-ventricular mass normalized to tibia length was similar between A-SED and A-ETR (a). Ejection fraction (EF,%; b), fractional shortening (FS, %; c), stroke volume (SV, μl; d), and cardiac output (CO, μl/min, e) were greater in A-ETR vs. A-SED mice, whereas passive diastolic filling (MVE, mm/s, f), active diastolic filling (MVA, mm/s, g), the MVA/MVE ratio (E/A, h), and diastolic filling pressure (E/e’, ratio; i) were not different between groups. (j) Representative images of blood flow velocity obtained during the assessment of MVE and MVA from both groups. The myocardial performance index (MPI, k), an indicator of overall LV function, was similar between A-ETR vs A-SED. For (a-i and k), n=8-9, *p<0.05 vs A-SED. Data are expressed as mean ± SEM.

**Supplementary Figure 13.** ***Cardiac function in trained and untrained adult mice.*** These data supplement main Figure 5. A mice did (ETR) or did not (SED) complete 12-weeks of treadmill-running. At least 24 h following the last exercise bout transthoracic echocardiography was completed. Mean data ± standard error are shown (a-d). (a) Left ventricular (LV) internal dimension in systole (LVIDs, mm) and (b) diastole (LVIDd, mm), and (c) end-systolic (ESV, µl) and (d) end-diastolic (EDV, µl) volume were similar in A-SED and A-ETR mice. For (a-d), n=8-9.

**Supplementary Figure 14.** ***Late-in-life exercise training improves antioxidant gene expression but not fibrosis in mouse hearts.*** These data supplement Figure 5. O mice did (ETR) or did not (SED) complete 12-weeks of treadmill-running. At least 24 h following transthoracic echocardiography, hearts were prepared as appropriate. (a) Representative immunohistochemistry images and mean data ± standard error indicate type 1 collagen (%, b) and cardiomyocyte cross-sectional area (μm^2^, c) were similar between groups. The scale bar represents 50 μm (top) and 20 μm (bottom). mRNA expression of fibrosis-related genes (d) and antioxidant genes (e) indicate : (i) no differences exist between groups concerning the profibrotic genes fibrillin (Fbn) 1 and 2, transforming growth factor-β (Tgfb) 1 and 2, and connective tissue growth factor (Ctgf) in hearts from O-ETR vs. O-SED mice. For (d) data are expressed as fold change relative to values of O-SED. For (b, c), n=6 mice per group, n=5 fields of view. For (d), n=5-9 per group.

**Supplementary Figure 15.** ***Ponceau staining loading control for assessment of OXPHOS complex.*** These data supplement main Figure 6. A representative image of complex I-IV of the electron transport chain is shown in Figure 6g. The above representative image shows Ponceau red staining from the same membrane. Histograms shown in main Figure 6 (h-l) are normalized to protein loading shown above.

**REFERENCES**

Bharath, L. P., Cho, J. M., Park, S.-K., Ruan, T., Li, Y., Mueller, R., . . . Symons, J. D. (2017). Endothelial Cell Autophagy Maintains Shear Stress-Induced Nitric Oxide Generation via Glycolysis-Dependent Purinergic Signaling to Endothelial Nitric Oxide Synthase. *Arteriosclerosis, thrombosis, and vascular biology, 37*(9), 1646-1656. doi:10.1161/ATVBAHA.117.309510

Bharath, L. P., Ruan, T., Li, Y., Ravindran, A., Wan, X., Nhan, J. K., . . . Symons, J. D. (2015). Ceramide-Initiated Protein Phosphatase 2A Activation Contributes to Arterial Dysfunction In Vivo. *Diabetes, 64*(11), 3914-3926. doi:10.2337/db15-0244

Dai, D. F., & Rabinovitch, P. S. (2009). Cardiac aging in mice and humans: the role of mitochondrial oxidative stress. *Trends Cardiovasc. Med, 19*(7), 213-220. doi:S1050-1738(09)00182-0 [pii];10.1016/j.tcm.2009.12.004 [doi]

Dai, D. F., Santana, L. F., Vermulst, M., Tomazela, D. M., Emond, M. J., MacCoss, M. J., . . . Rabinovitch, P. S. (2009). Overexpression of catalase targeted to mitochondria attenuates murine cardiac aging. *Circulation, 119*(21), 2789-2797. doi:CIRCULATIONAHA.108.822403 [pii];10.1161/CIRCULATIONAHA.108.822403 [doi]

DiMemmo, L. M., Cameron Varano, A., Haulenbeek, J., Liang, Y., Patel, K., Dukes, M. J., . . . Kelly, D. F. (2017). Real-time observation of protein aggregates in pharmaceutical formulations using liquid cell electron microscopy. *Lab Chip, 17*(2), 315-322. doi:10.1039/c6lc01160h

Erickson, P. A., Anderson, D. H., & Fisher, S. K. (1987). Use of Uranyl Acetate En-Bloc to Improve Tissue Preservation and Labeling for Postembedding Immunoelectron Microscopy. *Journal of Electron Microscopy Technique, 5*(4), 303-314. doi:DOI 10.1002/jemt.1060050403

Flynn, J. M., O'Leary, M. N., Zambataro, C. A., Academia, E. C., Presley, M. P., Garrett, B. J., . . . Melov, S. (2013). Late-life rapamycin treatment reverses age-related heart dysfunction. *Aging Cell, 12*(5), 851-862. doi:10.1111/acel.12109 [doi]

Goroshi, M., & Chand, D. (2016). Myocardial Performance Index (Tei Index): A simple tool to identify cardiac dysfunction in patients with diabetes mellitus. *Indian Heart J, 68*(1), 83-87. doi:S0019-4832(15)00248-5 [pii];10.1016/j.ihj.2015.06.022 [doi]

Gottlieb, R. A., Andres, A. M., Sin, J., & Taylor, D. P. (2015). Untangling autophagy measurements: all fluxed up. *Circ. Res, 116*(3), 504-514. doi:CIRCRESAHA.116.303787 [pii];10.1161/CIRCRESAHA.116.303787 [doi]

Ju, J. S., Varadhachary, A. S., Miller, S. E., & Weihl, C. C. (2010). Quantitation of "autophagic flux" in mature skeletal muscle. *Autophagy, 6*(7), 929-935. doi:10.4161/auto.6.7.12785

Laor, D., Sade, D., Shaham-Niv, S., Zaguri, D., Gartner, M., Basavalingappa, V., . . . Gazit, E. (2019). Fibril formation and therapeutic targeting of amyloid-like structures in a yeast model of adenine accumulation. *Nat Commun, 10*(1), 62. doi:10.1038/s41467-018-07966-5

Pires, K. M., Buffolo, M., Schaaf, C., David Symons, J., Cox, J., Abel, E. D., . . . Boudina, S. (2017). Activation of IGF-1 receptors and Akt signaling by systemic hyperinsulinemia contributes to cardiac hypertrophy but does not regulate cardiac autophagy in obese diabetic mice. *Journal of molecular and cellular cardiology, 113*, 39-50. doi:10.1016/j.yjmcc.2017.10.001

Symons, J. D., Hu, P., Yang, Y., Wang, X., Zhang, Q. J., Wende, A. R., . . . Litwin, S. E. (2011). Knockout of insulin receptors in cardiomyocytes attenuates coronary arterial dysfunction induced by pressure overload. *Am J Physiol Heart Circ Physiol, 300*(1), H374-381. doi:10.1152/ajpheart.01200.2009

Symons, J. D., Rendig, S. V., Stebbins, C. L., & Longhurst, J. C. (2000). Microvascular and myocardial contractile responses to ischemia: influence of exercise training. *J Appl Physiol (1985), 88*(2), 433-442. doi:10.1152/jappl.2000.88.2.433

Zhang, Q.-J., Holland, W. L., Wilson, L., Tanner, J. M., Kearns, D., Cahoon, J. M., . . . Symons, J. D. (2012). Ceramide mediates vascular dysfunction in diet-induced obesity by PP2A-mediated dephosphorylation of the eNOS-Akt complex. *Diabetes, 61*(7), 1848-1859. doi:10.2337/db11-1399

**Supplementary Table 1.** ***Mouse characteristics.*** Adult (A, 5 mo) and older (O, 21 mo) mice did (ETR) or did not (SED) complete 12-weeks of treadmill-running. ETR increased heart weight regardless of age. Random-fed blood glucose was lower in O-SED vs. A-SED mice. Statistical analyses were performed by a one-way ANOVA followed by Tukeys post-test. *p<0.05 vs. A-SED; #p<0.05 vs. O-SED

**Supplementary Table 2.** ***Primers used for qPCR analyses.***

**Supplementary Table 3.** ***Antibodies used for immunoblotting analyses.***

**Supplementary Table 4.** ***Other materials used in this study.***
